# Research on the BiSpikeNet classification model for tea plant pests and diseases

**DOI:** 10.3389/fpls.2026.1859819

**Published:** 2026-09-25

**Authors:** Yalin Hu, Shengbing Che, Mengge Yin, Jingqian Tang, Yuqi Li, Fangping Yin, Yongmin Liu

**Affiliations:** 1 School of Computer Science and Mathematics, Central South University of Forestry and Technology, Changsha, Hunan, China; 2 School of Electronic Information and Physics, Central South University of Forestry and Technology, Changsha, Hunan, China; 3 School of Computer Science and Engineering, Changsha University of Science and Technology, Changsha, Hunan, China; 4 School of Computer Science and Mathematics,Central South University of Forestry and Technology, Changsha, Hunan, China

**Keywords:** BiRealNet, BiSNN, BiSpikeNet, diagnostic, ResNet, SNNMultiShortcutBlock, tea pest and disease identification

## Abstract

**Background:**

Fast and accurate identification of tea pests and diseases with lightweight, efficient, and resource-friendly computation is a key challenge in smart tea gardens. While deep learning models like ResNet extract strong features, their high computational cost limits application. Conversely, lightweight models like BiRealNet reduce complexity but suffer from unclear intermediate features and weak discrimination.

**Methods:**

To address these limitations, we propose BiSpikeNet, a lightweight, high-efficiency classification model integrating Convolutional Neural Networks (CNNs) and Spiking Neural Networks (SNNs) under a “Diagnostic” design paradigm. We introduced an attention mechanism to enhance key feature representation and optimized residual structures. Furthermore, a cross-modal fusion framework was designed, combining a binary SNN meta-network (BiSNN) with a pulse-based residual block (SNNMultiShortcutBlock) to capture temporal and spatial features.

**Results:**

The proposed model achieved 92.30% accuracy with fewer parameters. With the cross-modal fusion framework, the accuracy improved to 99.92%. Compared with existing lightweight models such as TLDDM and LiSA-MobileNetV2, our method demonstrates superior performance with lower computational costs.

**Conclusion:**

Experimental results demonstrate that BiSpikeNet provides an effective balance between accuracy and efficiency, offering a robust solution for lightweight, resource-efficient tea pest and disease detection. Code is available at https://github.com/huyalin070-svg/BiSpikeNet.

## 1 Introduction

Tea is one of the world’s most significant cash crops, particularly in Asia and many regions of China. It serves not only as the primary ingredient for residents’ beverages but also as a source of income for hundreds of millions of people (Le et al., 2023). However, during tea cultivation, diseases such as tea grey leaf spot, tea brown leaf spot, tea red leaf spot, and tea leaf spot, alongside pests including tea aphids, tea green leafhoppers, and tea leafhoppers, severely threaten both tea yield and quality (Roy et al., 2024). These pests and diseases not only cause yield reductions but also inflict substantial economic losses, disrupting the tea industry chain and undermining sustainable agricultural development (Chen and Luo, 2025). Consequently, rapid and accurate detection of tea pests and diseases is crucial for effective prevention and control, safeguarding tea yield and quality, and advancing agricultural modernization.

Traditional tea leaf inspection methods primarily encompass manual inspection and mechanical screening techniques (Zhang et al., 2025b). Manual inspection typically relies on experienced workers visually examining tea leaves to determine their type and quality (Xue et al., 2023). Mechanical screening separates tea leaves of different sizes based on mesh aperture dimensions (Pruteanu et al., 2023). Furthermore, image processing techniques such as edge detection, threshold segmentation, and colour analysis are widely employed in tea leaf identification projects. These technologies enable automated recognition of tea leaf images to a certain extent (Zhang et al., 2023). Such approaches rely on image pre-processing, feature extraction, and classification algorithms, enhancing detection efficacy under specific conditions (Wang et al., 2025c). However, their dependence on manually designed features and limited generalisation capability constrain practical application (Wang et al., 2025d). For instance, conventional machine learning methods typically require complex, disease-specific pre-processing and segmentation steps, making it difficult to capture deep image patterns and thus limiting their scalability in agricultural production (Li et al., 2024).

In recent years, the rapid advancement of artificial intelligence and computer vision technologies has propelled the application of deep learning in crop pest and disease identification. Compared to traditional methods, deep learning models—particularly convolutional neural networks (CNNs)—can autonomously learn high-level semantic features from large-scale image datasets, thereby significantly enhancing the accuracy and efficiency of tea pest and disease classification (Rodrguez-Lira et al., 2024). CNNs possess robust feature representation and generalisation capabilities, enabling them to process complex agricultural scenarios and accurately identify disease patterns (Zhang et al., 2025a).Beyond agricultural tasks, deep learning has also been extensively explored for visual enhancement and complex data association in challenging environments, including underwater image enhancement via visual–textual fusion, self- organized strategies, and cascaded enhancement protocols, as well as graph-based representation learning for radar signal processing (Wang et al., 2026, 2024a, 2023; Li et al., 2026).

To further enhance the model’s performance in complex environments, attention mechanisms have been incorporated. This mechanism assists the model in focusing on disease-related areas, thereby improving classification accuracy (Duhan et al., 2024). For instance, spatial attention emphasises key regions within images to improve localisation and recognition (Lin et al., 2024); channel attention dynamically adjusts the importance of different feature channels to better represent disease characteristics (Sajitha et al., 2026).

Moreover, the Multi-Layer Perceptron (MLP), as a typical feedforward neural network architecture, demonstrates robust performance in feature mapping and non-linear modelling. Models such as the MLP- Mixer, proposed in recent years for visual tasks, have also introduced novel approaches to image feature modelling (Shen et al., 2026). Concurrently, Spiking Neural Networks (SNNs), representing the third generation of artificial neural networks, have garnered increasing attention. SNNs transmit information by simulating the spiking mechanism of biological neurons (Guo et al., 2023), offering advantages such as event-driven computation and low power consumption. They demonstrate significant application potential in embedded and edge computing scenarios, while also providing a novel research direction for designing lightweight models for identifying tea leaf pests and diseases.

Despite significant advances in deep learning for tea leaf disease detection, numerous challenges persist in practical deployment. Factors such as symptom overlap, lighting variations, and background interference frequently cause recognition bias (Zhong et al., 2025). Concurrently, excessive computational demands severely constrain the efficient and lightweight application of models. The core task of image detectionlies in accurately assigning pixels to target objects, achieving precise pixel-level segmentation (Rodriguez-Sanchez et al., 2025). To balance accuracy and efficiency, lightweight models have emerged as a research focus, with lightweight binary neural networks becoming the preferred solution (Wang et al., 2025e). These models substantially reduce computational overhead by constraining weights to either +1 or -1. A representative example is BiRealNet (He et al., 2024), which enhances representational capacity while maintaining the efficiency inherent in binary networks. However, BiRealNet exhibits notable limitations in complex real-world environments. Firstly, despite its lightweight design, the second residual block captures features inaccurately, while the third residual block fails to extract intermediate-level features of tea leaf pests and diseases with sufficient clarity. This results in progressively blurred features obtained by subsequent residual blocks, thereby weakening the model’s overall feature representation capability. On the other hand, during the binarisation feature extraction process, the model exhibits a tendency to prioritise local information such as edge textures, making it challenging to effectively capture subtle disease characteristics and their temporal variations. This constraint hinders improvements in the model’s recognition performance.

To address the aforementioned challenges, this paper proposes a novel binary spike network model, BiSpikeNet, designed to enhance perception capabilities regarding the overall structure of tea plants and diseased areas while retaining its lightweight advantages. This enables precise and robust disease identification in complex environments.

The main contributions of this paper may be summarised as follows:

1. A dataset on pests and diseases from the Anhua Tea Plantation in Hunan comprising seven categories of tea plant states and 8,099 images was constructed and adopted. A standardised training, validation, and testing workflow was established through stratified random partitioning to ensure the scientific rigour and reliability of model evaluation.
2. The core concept of this paper lies in proposing a “Diagnostic” model design paradigm, where in the visualisation and quantitative analysis of feature maps serve as the direct driving force for model structural optimization. Through in-depth analysis of the semantic distribution, response intensity, and information flow pathways within each module’s feature maps, this study precisely identifies critical bottlenecks and redundant regions within the architecture, thereby enabling targeted design adjustments. Attention mechanisms are introduced in critical regions to enhance feature selectivity, while computational units are streamlined along redundant pathways to improve information transmission efficiency. This closed-loop process of “observation-diagnosis-design” ensures that every structural adjustment is grounded in interpretable visual evidence, achieving a synergistic evolution between lightweight design and feature representation capability.
3. This paper implements lightweight optimization of the model architecture. Firstly, an attention mechanism is introduced within the second residual block to enhance the model’s selective expression capability for key characteristics of tea leaf pests and diseases. Secondly, by reducing the number of iterations in the third residual block, the feature propagation pathway is optimised, thereby improving the clarity of mid-level feature expression. This achieves an effective balance between a lightweight structure and robust feature extraction capabilities.
4. The BiSpikeNet model presented herein resolves the core challenge of spiking neural networks under ultra-low-bit quantisation through two key structural innovations: Firstly, the BiSNN module was designed to achieve efficient spiking-to-binary computation at the meta-network architecture level, substantially reducing computational and memory overhead; Secondly, it innovatively proposes theSNNMultiShortcutBlock module. By establishing multiple temporal shortcut connections, it provides stable pathways for spike information propagation across the temporal dimension, effectively mitigating the gradient decay and temporal instability issues inherent in deep binary SNN training. Building upon this foundation, the model undergoes further lightweight optimization and incorporates Adaptive-PLIF neurons alongside SpikeNorm to enhance dynamic adaptability, while employing PReLU activation to amplify temporal nonlinearity. This approach reduces computational complexity, with Params decreasing by 50% and 43.84% relative to ResNet and BiRealNet respectively. Exploration of ultra-low power technology integration at the foundational level within crop pest and disease recognition has provided novel insights for a comprehensive, integrated, and highly efficient intelligent paradigm.

## 2 Relevant work

In recent years, with the continuous advancement of deep learning models in the field of visual recognition, lightweight and high-efficiency approaches have become key research directions in model design (Shahadat and Maida, 2025). Particularly in agricultural intelligent monitoring and edge computing scenarios, the key to solving the problem lies in reducing model complexity while maintaining recognition accuracy. Although traditional deep convolutional neural networks possess powerful feature representation capabilities, their substantial floating-point parameters and high computational overhead limit practical deployment on resource-constrained devices. Therefore, structurally optimized binary neural network design for lightweight, efficient, and redundancy-free computation has become a major research focus and been widely adopted, owing to its ability to significantly reduce parameters and computational overhead while retaining strong feature representation capability.

### 2.1 Lightweight design

In the design of deep vision models, lightweight architecture has emerged as a significant research focus. ResNet achieves outstanding performance through the stacking of numerous convolutional layers with residual connections. However, its full-precision convolutional operations and high-dimensional feature channels result in substantial parameter scales and computational complexity, making it challenging to run in lightweight, resource-saving systems. To reduce computational burden, researchers typically pursue lightweight structural designs by decreasing network depth, compressing channel counts, or introducing convolutional or depthwise separable convolutions (Wang et al., 2025a). BiRealNet substantially reduces model parameters and inference power consumption while preserving the residual learning mechanism (He et al., 2024). However, relying solely on structural compression may compromise feature representation capabilities. Particularly in multi-class fine-grained recognition tasks, excessive lightweighting can lead to insufficient mid-level semantic expression, resulting in issues such as indistinct subject-background separation and lacklustre edge responses. To mitigate feature degradation caused by lightweighting, attention mechanisms are widely applied in lightweight models. Channel attention mechanisms assign differential weights to distinct feature channels, thereby enhancing key discriminative features. Spatial attention mechanisms, meanwhile, help highlight target regions while suppressing background noise. However, attention modules typically introduce additional parameters and computational overhead. If poorly designed, they may undermine the efficiency advantages of lightweight models. Furthermore, within the binary network framework, feature representations are inherently compressed, potentially limiting the modulation capabilities of attention mechanisms. Consequently, achieving an effective balance between lightweight design and attention enhancement—ensuring models maintain stable and clear featurerepresentation capabilities while preserving low computational complexity—remains a critical challenge in current lightweight binary network design.

### 2.2 Binarization for metaverse applications

Neural network binarisation constitutes an extreme quantisation strategy, whose core principle involves compressing weights and activations into the representation {-1, +1} to achieve exponential compression in both storage and computation (Nardini et al., 2025). Conventional binarisation methods typically employ the sign function for hard threshold mapping; however, this approach suffers from gradient discontinuity and optimization difficulties during training, readily leading to performance degradation (Bulat et al., 2021). To address this issue, BiRealNet introduces a meta-network-based quantisation mechanism utilising a Multi- Layer Perceptron (MLP). This approach models the weight binarisation process as a learnable function. Compared to fixed-threshold quantisation, the meta-network dynamically learns optimal quantisation boundaries based on data distribution, thereby enhancing the adaptive capability of the quantisation process. This method mitigates the training instability inherent in traditional binarised networks to some extent and improves model recognition performance in complex visual scenarios. However, binarisation inherently constitutes a strong information compression process, retaining only sign information while significantly reducing numerical expressiveness. Even with the adaptive learning provided by the meta- network, it remains challenging to fully compensate for the fine-grained expressive capabilities inherent in floating-point weights. In complex backgrounds or regions with subtle textural variations, binary weights may struggle to generate highly discriminative feature responses. Furthermore, the meta-network itself introduces additional structure. Although its parameter scale is relatively small, it still introduces a degree of structural complexity in extreme lightweight scenarios. Simultaneously, the gradient approximation of the quantisation function remains reliant on surrogate gradient strategies. If poorly designed, this may lead to training oscillations or reduced convergence speed. Therefore, how to further enhance feature expressiveness and training stability while preserving the efficient inference advantages of binarisation remains a crucial direction for the continuous optimization of binary networks.

### 2.3 Residual block

The residual learning framework, proposed by He et al., centres on mitigating gradient vanishing and decay in deep networks through identity mappings, enabling networks to learn residual representations rather than full mappings (He et al., 2016). Within floating-point networks, residual structures have been demonstrated to significantly enhance training stability and feature representation capabilities in deep models.

BiRealNet constructs an enhanced binary residual block. This architecture integrates binary input streams, meta-network weight quantisation mechanisms, and efficient binary convolutional operations to achieve feature propagation and representation within a lightweight inference framework. However, within a binary environment, the effectiveness of residual structures remains constrained by quantisation precision limitations. Firstly, both the main branch and shortcut branch employ binary feature representations, whose expressive power is markedly restricted compared to floating-point networks. When intermediate features fail to sufficiently separate subjects from backgrounds, residual stacking may inadequately enhance discriminative information, potentially preserving redundant responses. Secondly, during multi- layer residual stacking, limited feature expressiveness across layers may yield suboptimal information accumulation, leading to edge blurring or response confusion in certain intermediate feature maps. Moreover, while reducing residual block counts for further lightweighting lowers computational costs, it may compromise feature hierarchical progression and semantic refinement capabilities, thereby affectingthe model’s robustness in complex scenarios. Overall, binary residual blocks inherit the advantages of residual learning while achieving efficient inference and low-power operation. However, there remains room for improvement in terms of fine-grained feature representation and background suppression capabilities.

### 2.4 Feature map analysis of the baseline model

From an architectural perspective, BiRealNet builds upon ResNet’s residual learning advantages. Through the synergistic design of the three key technologies outlined above, it achieves an effective balance between model expressiveness and computational efficiency, as illustrated in **Figure 1**. Building upon this structural design, the multi-layer feature maps in **Figure 1a** reveal the gradual evolution of features as the network deepens. At shallow layers, the extraction primarily focuses on local texture and grey-scale gradient information. Contrast between tea leaves and background begins to emerge, with more pronounced responses appearing in edge regions, though the overall emphasis remains on low-level detail features. At the intermediate layer stage, the tea leaves’ contours gradually sharpen, while irrelevant background information diminishes. The model begins to focus more intently on the primary structure of the tea leaves, particularly the outer leaf contours and certain damaged edge regions. This demonstrates that the binary convolutions in **Figure 1b** can, to a certain extent, preserve key shape information while reducing computational complexity. However, as visible in the feature maps corresponding to the second and third residual modules in **Figure 1a**, some degree of co-occurrence between tea leaves and background persists. Certain background regions retain strong responses, edge transitions lack sharpness, and the discernibility of tea leaf contours appears relatively blurred compared to preceding stages. At deeper stages, features undergo further abstraction, background interference is further suppressed, the overall tea leaf morphology becomes more concentrated, and edge structures exhibit relatively continuous high-response regions, demonstrating stable structural representation capabilities. Overall, this baseline model achieves a layer-by-layer transition from low-level textures to high-level structural features, demonstrating some capability in identifying tea leaf edges. However, in certain intermediate feature maps, the distinction between the subject and background remains insufficient. These observations provide a reference basis for subsequent analysis and model comparisons.

**Figure 1:**
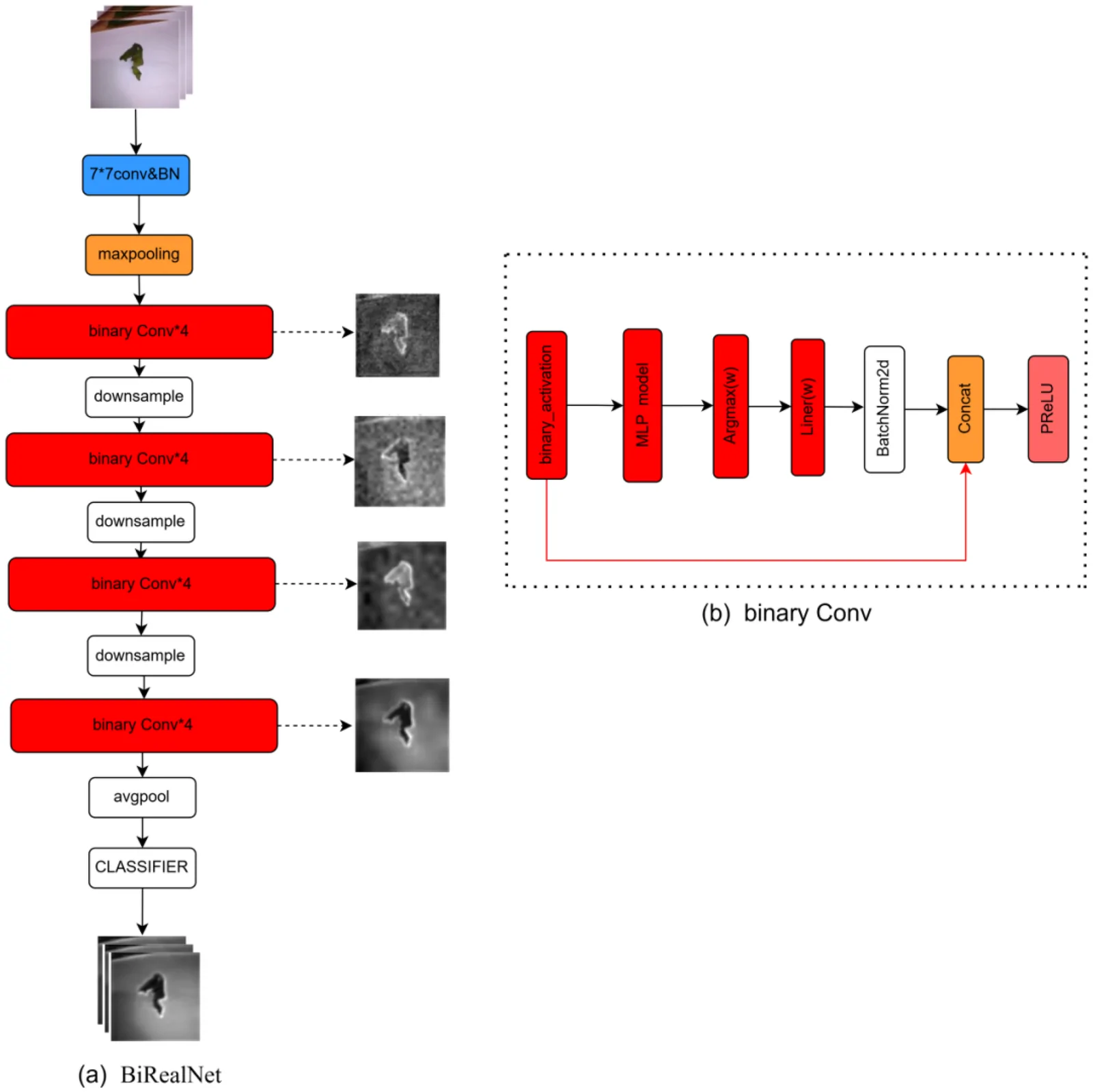
Overall model diagram of BiRealNet. **(a)** BiRealNet. **(b)** binary Conv.

## 3 Methodology

### 3.1 Data collection

The dataset utilised in this study was collected from multiple tea gardens within the Anhua region, Hunan Province, China, comprising 8,099 RGB images (2670 *×* 1200 pixels) categorised into seven tea leaf conditions: damaged tea leaves, tea mosquito blight, tea leaf brown spot disease, tea leaf red spot disease, tea leaf grey spot disease, tea leaf spot disease, and healthy tea leaves, as illustrated in **Figure 2**. These categories were defined based on actual field conditions in local tea production and reflect typical pest and disease patterns observed in Chinese tea plantations.

**Figure 2:**
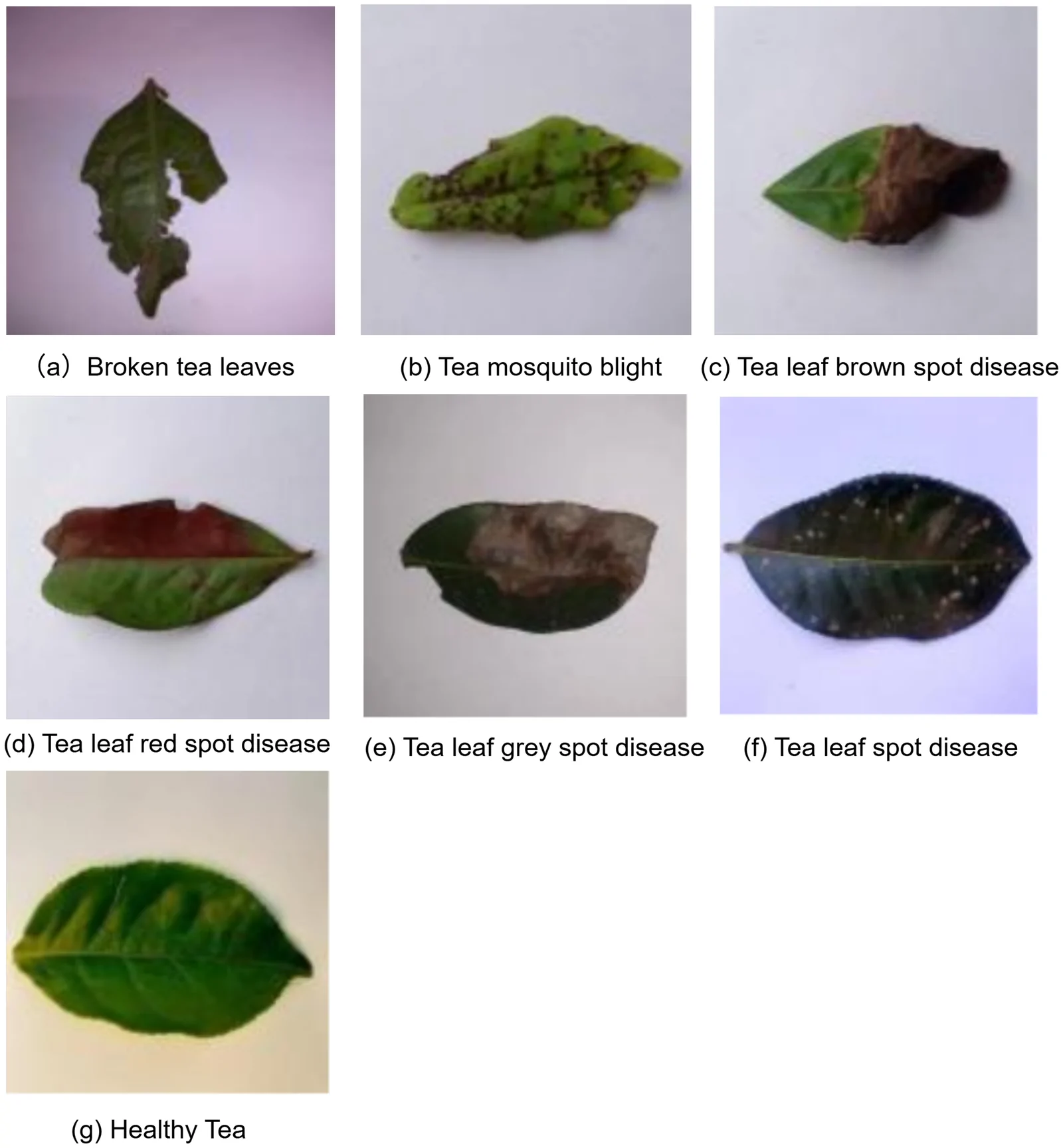
Categories of tea pest and disease dataset. **(a)** Broken tea leaves. **(b)** Tea mosquito blight. **(c)** Tea leaf brown spot disease. **(d)** Tea leaf red spot disease. **(e)** Tea leaf grey spot disease. **(f)** Tea leaf spot disease. **(g)** Healthy Tea.

To address class imbalance, a random oversampling strategy was employed by directly replicating samples from minority classes according to predefined coefficients, thereby ensuring a more balanced distribution among categories during model training. It is important to emphasise that tea pest and disease datasets inherently exhibit strong regional characteristics. For example, both the publicly available Tea Leaf Disease Dataset and TeaLeafBD, which were collected in Bangladesh and released on the Mendeley Data platform, define disease categories according to local tea cultivation conditions and regional disease characteristics. To further evaluate the robustness and generalisation capability of the proposed model, additional experiments were conducted by training and testing on the publicly available Tea Leaf Disease Dataset. The model achieved an average classification accuracy of 98%, demonstrating that the proposed approach maintains strong performance on independent public datasets and therefore possesses a certain degree of generalisation ability.

In contrast, the present study focuses specifically on tea gardens in China, and the category definitions are tailored to the regional agronomic environment of Anhua. The dataset contains samples collected under different lighting conditions and complex background scenarios, thereby reflecting the diversity and variability encountered in real tea plantation environments. As one of the representative tea-producing regions in China, Anhua provides highly representative field conditions for tea pest and disease recognition research. The classification system was developed based on extensive field experience and further validated by professional tea masters, nationally recognised tea plantation experts, and senior horticulturists with more than 20 years of experience. In addition, the study is supported by a national-level tea plantation and a university-affiliated training base.

For data partitioning, a stratified random sampling strategy was adopted, with 10% of the dataset used as the test set for final performance evaluation. The remaining 90% was further split into a training subset (80%) and a validation subset (20%) for model development and evaluation. The training subset facilitatedmodel fitting and parameter optimization, while the validation subset guided hyperparameter tuning and monitored training progress. The sample distribution across each subset is detailed in **Table 1**.

**Table 1:** Classification of tea pests and diseases.

Class	Training set	Validation set	Test set	Oversampling coefficient
Broken tea leaves	416	87	23	2.51
Tea mosquito blight	881	205	119	1.19
Tea leaf brown spot disease	914	184	68	1.14
Tea leaf red spot disease	1046	233	97	–
Tea leaf grey spot disease	903	210	97	1.16
Tea leaf spot disease	1019	218	94	1.03
Healthy Tea	962	211	112	1.09

### 3.2 Dataset

As shown in **Table 1**, the dataset employed in this study exhibits significant category imbalance. Certain disease categories possess substantially more training samples than others: tea leaf red spot disease, tea leafgrey spot disease, tea mosquito blight, and tea leaf spot disease comprise 1,143, 1,000, 1,000, and 1,113 images respectively, constituting the majority categories with larger sample sizes. In contrast, the damaged tea leaves category possesses the smallest sample size, comprising merely 439 images; the brown spot disease category also exhibits relatively limited samples, totalling 980 images. Both categories fall markedly below the overall average sample size. Such imbalanced distribution poses potential challenges during model training. Minority categories may fail to acquire sufficient feature representations during training, leading to diminished model recognition capabilities for these categories and consequently impacting overall classification performance and generalisation ability.

To mitigate the adverse effects of class imbalance, this study employs an oversampling-based class balancing strategy. Oversampling is one of the commonly used techniques for addressing class imbalance, with primary methods including random oversampling (He and Garcia, 2009) and synthetic oversampling techniques (Bunkhumpornpat et al., 2024). In this work, we replicate minority class samples according to a predefined ratio to achieve balanced distribution with the majority class within the training set (see **Table 1** for details). By increasing the frequency of minority class samples in training batches, this strategy enables the model to learn their discriminative features more thoroughly during optimization, thereby enhancing classification sensitivity towards the minority class.

To further enhance the diversity of oversampled training samples and bolster model robustness, we concurrently introduce multiple geometric spatial transformations during oversampling to constitute a data augmentation pipeline. Specific augmentation operations comprise: random rotation (within an angular range of ±10°), translation (with amplitude of ±10% relative to image dimensions), scaling (with proportional variation of ±5%), and horizontal and vertical mirror filling. These enhancements are generated in real-time during training, effectively simulating perspective variations, scale discrepancies, and orientation uncertainties inherent in real-world agricultural image acquisition. This guides the model to learn key feature representations exhibiting rotational and scale invariance. This approach not only mitigates overfitting risks stemming from class imbalance to a certain extent but also enhances the model’s adaptability within complex field environments. Importantly, dataset splitting into training, validation, and test sets was performed before oversampling and augmentation, and these operations were applied exclusively to the training set to prevent data leakage.

### 3.3 Development of the BiSpikeNet model

Research indicates that binarisation strategies can significantly reduce model parameter size and computational complexity, serving as an effective approach for achieving efficient edge deployment. However, in practical applications, relying solely on weight and activation binarisation often diminishes feature representation capabilities. This is particularly evident in complex backgrounds or fine-grained category recognition tasks, where models may struggle with inadequate subject-background separation and insufficient edge information representation. Consequently, the core challenge addressed by this paper’s methodology is how to enhance feature modelling capabilities while preserving the efficiency of binary networks.

Among existing lightweight binary networks, BiRealNet achieves high inference efficiency by combining a residual learning framework with binary convolution operations. This architecture utilizes a binary input stream, an MLP-driven adaptive weight quantization mechanism, and XNOR-bitcount computation units to maintain reasonable recognition accuracy while ensuring low-power operation. However, compressing feature representations into the {-1, +1} format limits the network’s semantic expressiveness in intermediate layers. This may result in scattered object responses and residual background noise in feature maps.Furthermore, reducing the number of layers to meet lightweight requirements further constrains the feature abstraction capability at each layer.

To address the aforementioned issues, this paper proposes a novel binary spike neural network model, named BiSpikeNet. Building upon the principles of residual learning frameworks and efficient binary convolutions, it reconstructs and streamlines key residual modules and feature propagation pathways. This approach enhances mid-level semantic representation capabilities while maintaining an overall lightweight structure. Concurrently, a novel meta-network SNN-driven binarisation and feature modulation mechanism amplifies response intensity in critical channels and primary regions while suppressing background interference. This collaborative design effectively mitigates limitations arising from lightweight structures, information compression in meta-network binarisation, and insufficient feature aggregation in residual blocks. It achieves stable and comprehensive tea leaf structure recognition while preserving high-efficiency inference capabilities.

#### 3.3.1 Lightweight module LB-BiRealNet

To more effectively extract key features from images of tea plant pests and diseases, this paper undertakes targeted module optimization and structural adjustments to the BiRealNet model. The core design comprises the following two aspects.

Firstly, given that tea disease images typically exhibit locally prominent features and moderate complexity, this paper streamlines the number of residual blocks within the model. The total residual blocks are reduced from 16 to 14, achieved specifically by decreasing the iteration count of the third residual block by two cycles. This adjustment significantly reduces the parameter size and computational burden while preserving the model’s representational capacity. This characteristic renders the model more suitable for lightweight, resource-efficient practical scenarios with strict computation limits.

Secondly, to enhance the second residual block’s ability to focus on key disease-affected regions, this study introduces a lightweight and efficient channel attention mechanism: the ECA module (Shrisha and Anupama, 2025). This module is embedded within the residual network architecture. The ECA module can adaptively calibrate channel feature responses with minimal computational overhead. This mechanism effectively improves the second residual block’s perception of subtle disease features and its capacity for discriminative representation learning.

As illustrated in **Figure 3**, within the integrated LB-BiRealNet architecture, the evolution of features across network layers can be observed from the multi-layer feature maps depicted in **Figure 3a**. At shallow stages, the representation remains dominated by local texture and grey-scale gradient information, with initial contrast emerging between tea leaves and background. Preliminary responses appear in edge regions, though overall feature expression remains biased towards low-level details. Upon reaching the intermediate layers, tea leaf contours gradually sharpen while background-irrelevant information diminishes. The network increasingly focuses on the principal leaf structure, particularly where leaf margin outlines and local defect regions commence forming relatively continuous high-response zones. Compared to the baseline model, the feature map corresponding to the third module in **Figure 3a** after convolution in the module of **Figure 3c** exhibits noticeable changes: the previously observed background-leaf blending is mitigated, with significantly reduced response intensity in background regions and more uniform grey-scale distribution, while activation becomes more concentrated within the leaf body areas. The leaf edges, previously somewhat blurred with indistinct transitions, now exhibit clearer boundaries and more pronounced contrast in structural representation. The main tea leaf contours appear more complete, with significantly enhanced shape recognition. At deeper stages, features are further abstracted as theypass through **Figure 3b**, background interference is continuously suppressed, and the overall tea leaf morphology presents a concentrated and continuous high-response distribution, resulting in more stable structural representation. From the perspective of overall feature evolution, the redesigned model maintains its lightweight characteristics while enhancing the representation of the tea leaf itself at intermediate stages. Particularly within the third module, it achieves a transition from “strong background response with insufficient subject clarity” to “prominent subject areas with distinct edge structures”. This transformation significantly strengthens the model’s ability to localise and identify areas affected by tea leaf pests and diseases.

**Figure 3:**
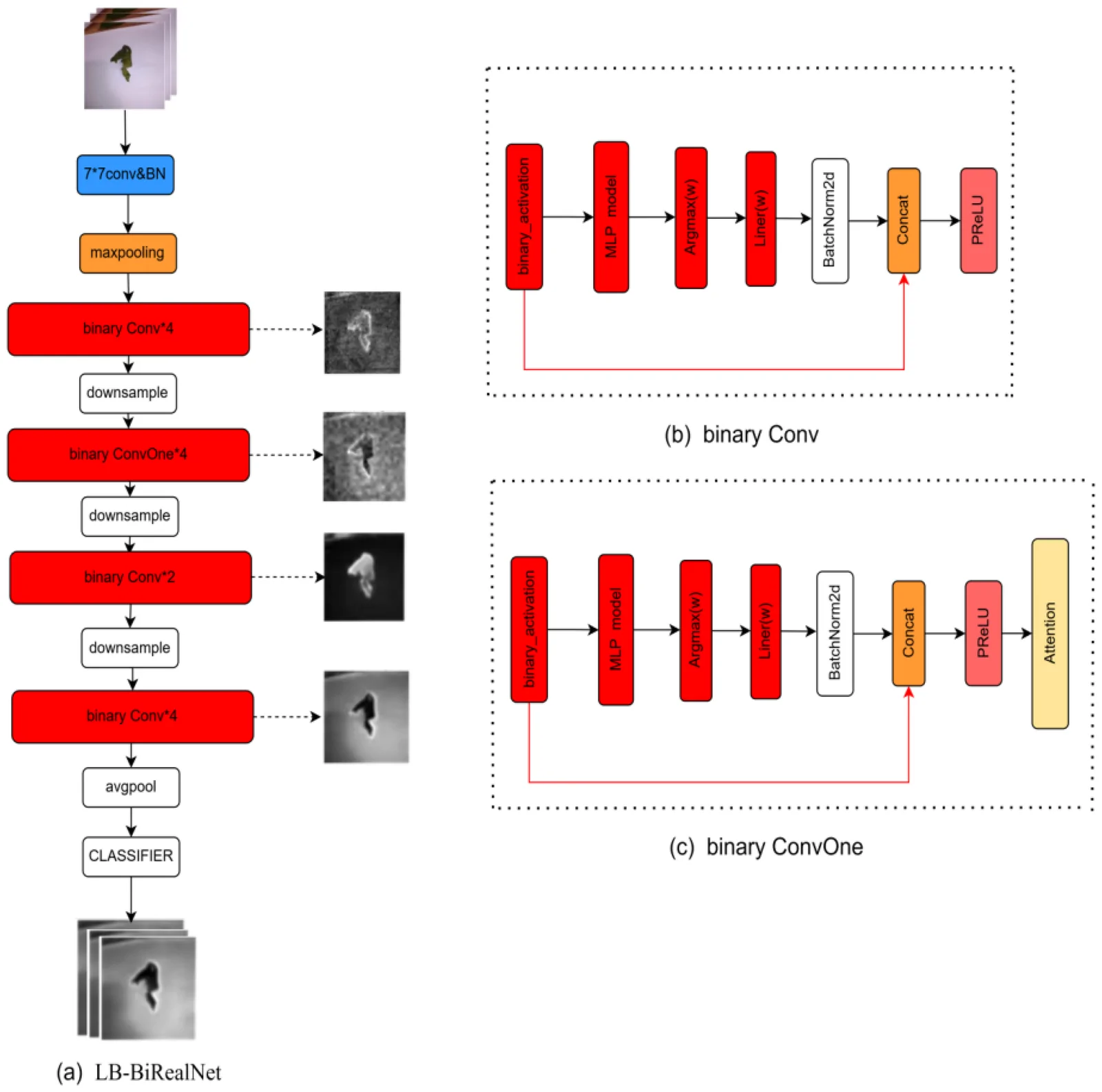
Overall model diagram of LB-BiRealNet. **(a)** LB-BiRealNet. **(b)** binary Conv. **(c)** binary ConvOne.

Although this structure demonstrates relatively pronounced edge response and possesses a certain capacity for delineating tea leaf contours, it remains deficient in overall regional modelling. The feature map exhibits relatively dispersed representation of the tea leaf’s interior regions, with insufficiently concentrated structural responses. Consequently, the network relies more heavily on edge information for classification, resulting in a relatively vague depiction of the tea leaf’s complete morphology and internal semantic features. This tendency to emphasise local boundaries while weakening the expression of overall structure limits the model’s comprehensive understanding of lesion distribution and overall morphological variations to some extent. It also provides theoretical motivation for proposing subsequent structural optimization models.

#### 3.3.2 Design of the BiSNN metanetwork

In this paper, we design the meta-network for convolutional operations based on a binary-based strategy for SNNs, as illustrated in **Figure 4c**.

**Figure 4:**
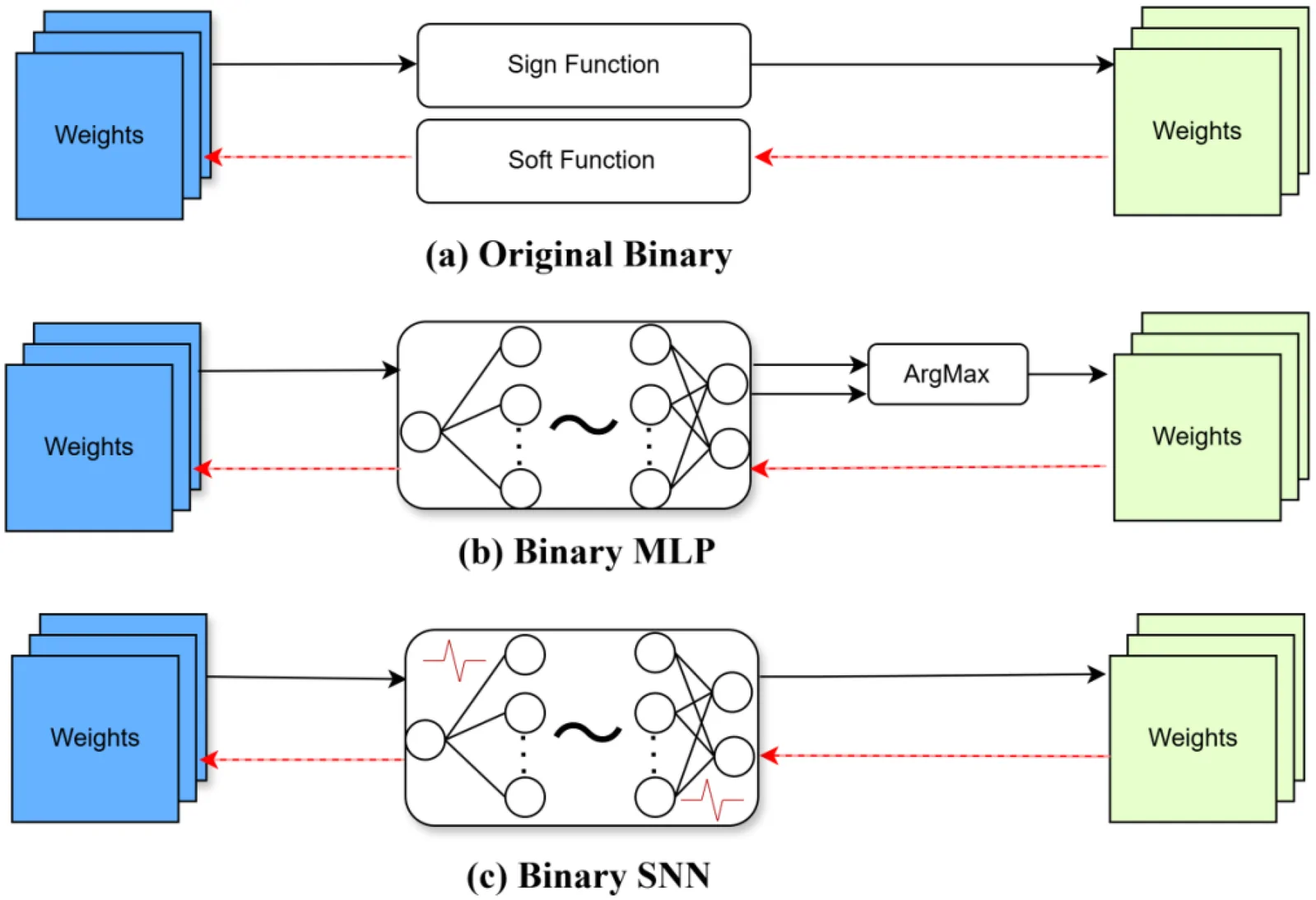
Binarised forward and backward propagation methods. **(a)** Original binary. **(b)** Binary Mlp. **(c)** Binary SNN.

The traditional meta-network MLP binarisation module depicted in **Figure 4b** employs a fully connected network to perform binary classification predictions on weights. Whilst this mechanism mitigates the gradient discontinuity issue introduced by the sign function to some extent, its binarisation process remains reliant on static mapping, necessitating a discretisation step to convert the binarised weights. This approach lacks dynamic expression across the temporal dimension. Furthermore, the hard-boundary classification inherent to MLP tends to cause excessive concentration of weight gradients during training, thereby constraining the model’s ability to distinguish complex features (Zhao et al., 2025).

To overcome this limitation, this paper proposes a novel design approach employing SNN pulse dynamics for weight quantisation. An Adaptive Parametric LIF (Adaptive-PLIF) neuron is introduced into the meta- network BiSNN as the core neuron for SNN binarisation. The membrane potential evolution of the Adaptive- PLIF neuron is described as follows: the membrane potential accumulates over time upon receiving input pulses. Upon reaching a preset threshold, the neuron fires a pulse and resets the membrane potential. Concurrently, adaptive parameters regulate the leakage and recovery rates of the membrane potential, enabling the neuron to dynamically adjust its firing characteristics based on input. This mechanism endows the weight quantisation process with temporal dynamic expression capabilities, effectively mitigating the issue of gradient convergence in binarisation while enhancing the model’s discriminative learning ability for subtle features of tea leaf pests and diseases.

The membrane potential update process of the spiking neuron is defined as shown in Equation 1:

(1) $$V_{t}=\alpha V_{t-1}\;+I_{t}$$

Where *Vt* denotes the membrane potential at time step *t*, *Vt—*1 represents the membrane potential at the previous time step, *α* is the learnable membrane potential decay coefficient, and *It* is the input current at time step *t*.

A spike is generated when the membrane potential exceeds the learnable threshold *v* th, as described in Equation 2:

(2) $$S_{t}=I(V_{t}\geq v_{\text{th}})$$

where *St* denotes the spike output at time step *t*. I() is the indicator function, and *v* th is the learnable firing threshold.

Since the indicator function in Equation 2 is non-differentiable, a triangular surrogate gradient is adopted during backpropagation to approximate its derivative while preserving binary spike generation in the forward pass. The surrogate gradient is defined in Equation 3:

(3) $$\frac{\partial S_{t}}{\partial V_{t}}\approx \frac{1}{k}\max(0,\;1-|\frac{V_{t}-v_{\text{th}}}{k}|)$$

where *k* controls the width of the surrogate gradient region. As shown in Equation 3, non-zero gradients are maintained within a neighbourhood of the firing threshold, enabling effective gradient propagation and stable optimization of the Adaptive-PLIF neurons, thereby improving convergence and training stability. Compared to the static binary thresholding used in MLPs, the thresholding mechanism of SNNs operates through a cross-temporal spike accumulation process, as described in Equations 1 and 2, enabling dynamic, continuous, and memory-based thresholding rather than single-step discrimination. This membrane potential-based integration mechanism results in smoother threshold transitions, which helps mitigate training instability caused by steep gradient changes while preserving richer gradient information during backpropagation.

Additionally, this study introduces a learnable scale factor to perform soft-boundary modulation on the final spike output, as shown in Equation 4:

(4) $$\tilde{S}=\tanh(\gamma \times (2S_{t}\_1))*0.5$$

where *γ* is a learnable modulation factor that controls the modulation intensity, and $\tilde{S}$ is the final modulated spike output. Compared to the hard transitions produced by the traditional sign function, this soft modulation method provides smoother binary gradients, which helps improve convergence stability and generalization capability.

Ultimately, the SNN binarization module demonstrates superior propagability compared to traditional MLP binary classifiers through dynamic weight discrimination in the temporal dimension and spike-based soft modulation. This module achieves more flexible binary representation capabilities, as illustrated in **Figure 4**. This design not only enhances binarization accuracy but also improves the model’s adaptability to complex visual patterns. Consequently, BiSpikeNet achieves a more stable training process and higher classification performance under binarization conditions.

#### 3.3.3 Design of the SNNMultiShortcutBlock pulse module

After completing the design of the binary mechanism at the meta-network level, this paper designed the SNNMultiShortcutBlock as the spike-residual structure for BiSpikeNet to complement BiSNN, thereby implementing a “spike-residual” collaborative mechanism. This structure achieves multi-path feature fusion through three binary convolutional branches (MetaConv2d):

(5) $$F=F_{1}+F_{2}+F_{3}$$

As shown in Equation 5, *F* denotes the final aggregated feature, while *F*_1_, *F*_2_ and *F*_3_ represent the feature outputs of the three parallel branches, respectively. Each sub-branch incorporates pulse-binary activation, pulse-binary convolution, and batch normalization operations. The multi-branch architecture significantly enhances the diversity of pulse-binary features by aggregating information from different branches, thereby strengthening the representational capacity of pulse-binary networks under the “information bottleneck” constraint.

Additionally, three key enhancement mechanisms have been incorporated into the SNNMultiShortcutBlock:

1. Adaptive-PLIF Pulse Neuron: Used for temporal pulse integration of multi-branch outputs, enabling residual mapping to exhibit dynamic temporal characteristics.
2. SpikeNorm: Performs normalization and steady-state adjustment of spikes along the temporal dimension to enhance the balance of spike firing distributions, preventing overly sparse or dense binary activations.
3. Attention: Used to capture positional dependencies, it restores more fine-grained spatial features under binary conditions, helping to reduce representation loss caused by binarization.

Ultimately, the residual between the fused output and the initial output is added, followed by a nonlinear transformation via PReLU to achieve enhanced nonlinear modelling capabilities. The overall model architecture significantly enriches feature representation while maintaining minimal computational cost, as illustrated in **Figure 5**. As evident in **Figure 5a**, following convolutions in **Figures 5b, c**), the feature maps consistently delineate the tea leaf’s primary contours. Edge regions exhibit continuous high-response distributions, while internal diseased areas begin displaying discernible brightness variations. This demonstrates the network’s emerging capacity for jointly representing shape and localised anomalous regions. In the original architecture, the third residual block, due to the superimposition of strong temporal pulse information (continuous modulation from Adaptive-PLIF and SpikeNorm), exhibits a diffusion effect in the spatial dimension. This results in a generalised reduction in contrast and softened edges within the output feature maps. It can be observed that the feature response becomes diffuse, the boundary between the tea leaves and the background becomes less sharp, local details are smoothed to some extent, and the overall appearance becomes somewhat blurred. In **Figure 5a**, directly removing this third residual block simplifies the feature transmission path and weakens the temporal accumulation effect. The final feature map output in **Figure 5a** clearly shows a more complete and distinct overall tea leaf morphology, with concentrated responses in the main area and effectively suppressed background regions. Compared to the models in **Figures 2a** and **3a**, the leaf edges appear sharper with improved contour continuity. Simultaneously, the contrast in details within pest-damaged areas becomes more pronounced. Identification no longer relies solely on edge responses but instead forms a clearer spatial representation of the tea leaves as a whole and their internal anomalous structures. The final fused feature map exhibits a more concentrated brightness distribution, with distinct separation between subject and background. Its overall clarity surpasses the aforementioned reference structures, demonstrating more stable spatial structure modelling capabilities.

**Figure 5:**
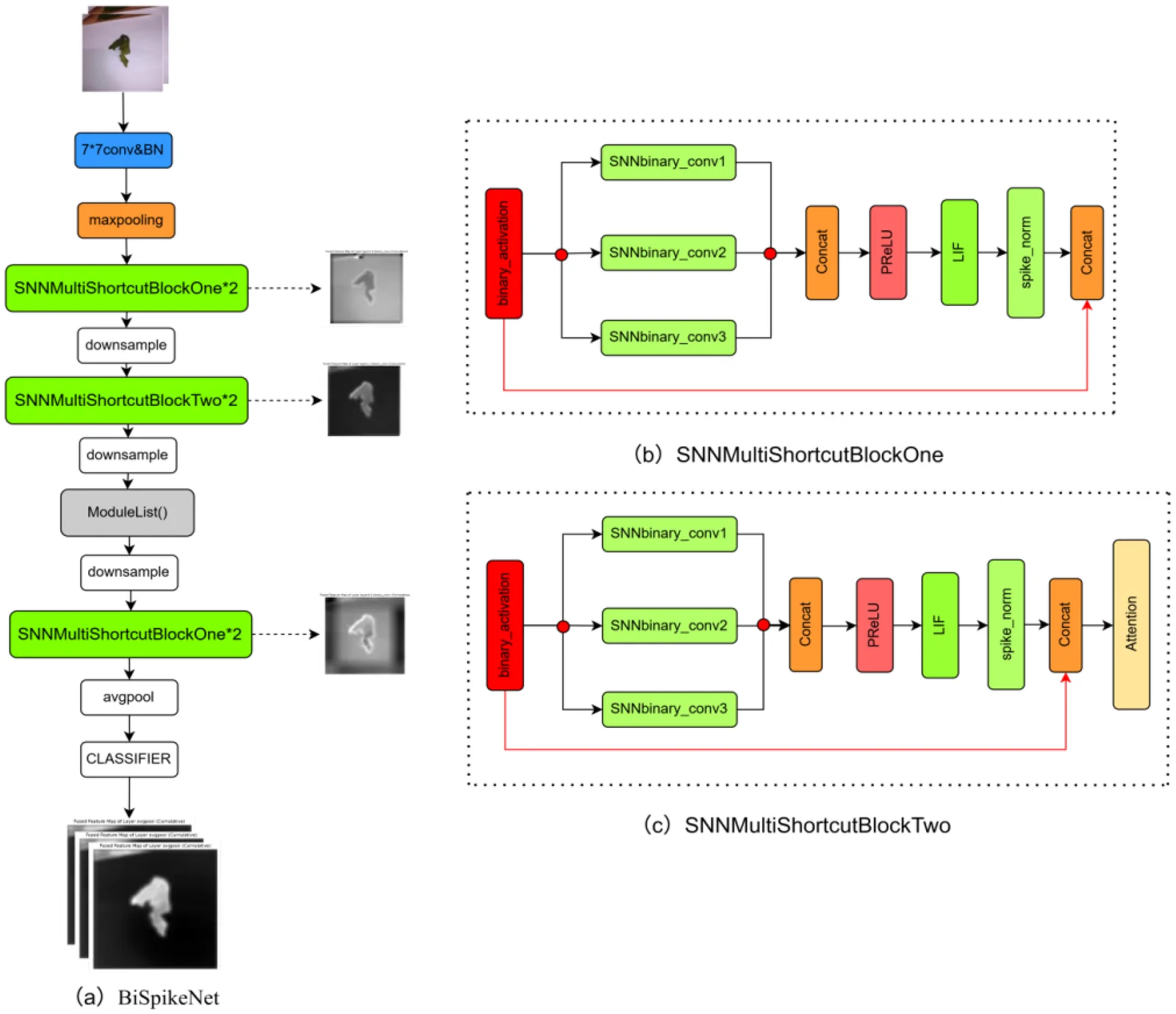
Schematic diagram of the BiSpikeNet model. **(a)** BiSpikeNet. **(b)** SNNMultiShortcutBlockOne. **(c)** SNNMultiShortcutBlockTwo.

#### 3.3.4 Model design overview

To systematically evaluate the individual contributions and combined effects of reconstruction via binarization, pulse dynamics enhancement, and attention mechanisms within a binary convolutional architecture, this paper designs and compares four variants. Each variant is described as follows.

##### 3.3.4.1 LB-BiRealNet

This variant adopts a simplified BiRealNet architecture, reducing the number of residual blocks while incorporating attention mechanisms on appropriate residual blocks. It retains the original MLP binarization and binarized convolutional functions.

##### 3.3.4.2 BiSNNNet

This model builds upon LB-BiRealNet by designing the meta-network as an SNN temporal binarization mechanism. It employs Adaptive-PLIF neurons to achieve dynamic binary classification across timesteps, eliminating the need for subsequent discretization after binarization, unlike MLP approaches. This modification aims to mitigate the gradient clipping issues inherent in static binarization. Since spike activation is non-differentiable, a surrogate gradient strategy is adopted during backpropagation while preserving binary spike generation in the forward pass, thereby enabling smoother and more stable weight binarization. Consequently, it improves convergence speed and generalization performance.

##### 3.3.4.3 BiMultiNet

In this variant, the SNNMultiShortcutBlock pulse residual block was designed. This module achieves multi-path feature fusion through three-branch binary convolutions. Simultaneously, it enhances the feature expression capability of the binary network under low-bit conditions via cross-temporal processing by Adaptive-PLIF neurons and SpikeNorm for pulse steady-state regulation. While maintaining other structures unchanged, it evaluates the improvement effect brought by “replacing only the residual structure” to the network.

##### 3.3.4.4 BiSpikeNet

This model integrates the three aforementioned design categories:

1. Structure-level lightweight design to reduce computational complexity while preserving expressive power.
2. The appropriate incorporation of attention mechanisms serves to enhance the spatial representation of binary features.
3. SNN temporal-based binarization replaces MLP binarization using Adaptive-PLIF neurons.
4. SNNMultiShortcutBlock has emerged as the new residual architecture, enabling multi-path feature fusion through ternary binary convolutions.

This variant combines the advantages of structural lightweighting, meta-network binarisation, and pulse residual block stabilisation. It constitutes the complete design version proposed in this study, aiming to maximise the feature representation capability and overall accuracy of the binary network.

### 3.4 Model evaluation

To evaluate the performance of the BiSpikeNet model in tea pest and disease identification tasks, this study employs several commonly used evaluation metrics. These metrics include accuracy, precision, recall, F1 score, floating-point operations per second (FLOPs), and number of parameters. Each metric assesses model performance from different dimensions.

Accuracy measures the proportion of correctly classified samples out of all samples, as shown in Equation 6. Precision evaluates the proportion of true positives among samples predicted as positive by the model, as shown in Equation 7. Recall measures the proportion of actual positive samples correctly identified by the model, as shown in Equation 8. The F1 score is the harmonic mean of precision and recall, providing a balanced evaluation when both false positives and false negatives require consideration, as shown in Equation 9. FLOPs quantifies the computational cost of a model by calculating the number of floating-point operations in a single forward pass, as shown in Equation 10. The number of parameters reflects the total number of trainable parameters in the model, indicating its memory usage and structuralcomplexity.

(6) $$\text{Accuracy}=\frac{\sum_{i=1}^{k}{\text{TP}}_{i}}{N}$$

(7) $$\text{Precision}=\frac{\text{TP}}{\text{TP}+\text{FP}}$$

(8) $$\text{Recall}=\frac{\text{TP}}{\text{TP}+\text{FN}}$$

(9) $$\text{F}1-\text{score}=\frac{2\;\times \text{Precision}\times \text{Recall}}{\text{Precision}+\text{Recall}}$$

(10) $$\text{FLOPs}=2\times C_{\text{in}}\times C_{\text{out}}\times K_{\text{h}}\times K_{\text{w}}\times H_{\text{out}}\times \;W_{\text{out}}$$

Among these metrics, TP (True Positive): the number of correctly predicted positive samples; TN (True Negative): the number of correctly predicted negative samples; FP (False Positive): Number of negative samples incorrectly classified as positive; FN (False Negative): Number of positive samples incorrectly classified as negative. *C*in and *C*out: Number of input and output channels, respectively. *K*h and *K*w: Height and width of the convolutional kernel; *H*out and *W*out: Height and width of the output feature map.

## 4 Experimental results and analysis

### 4.1 Experimental environment

All experiments were conducted on a system running Microsoft Windows 11, equipped with an Intel Core i5-12400F CPU and an NVIDIA GeForce RTX 4070 graphics processing unit (12 GB VRAM). The software environment comprised Python 3.11, PyTorch 2.7.0, CUDA 12.6, and cuDNN 9.1.

Training was performed for 150 epochs with a batch size of 32 using the Adam optimiser with an initial learning rate of 2.5 × 10ˆ-3. A linear learning rate decay strategy was adopted using a LambdaLR scheduler to gradually reduce the learning rate over the training process. A knowledge distillation strategy was employed to improve the performance of the lightweight spiking neural network. A pretrained ResNet- 18 model was adopted as the teacher network, while BiSpikeNet served as the student network in the knowledge distillation framework. The overall training objective consists of a weighted combination of the standard cross-entropy loss and the Kullback–Leibler (KL) divergence between the softened output distributions of the teacher and student networks. A distillation temperature of T = 3.0 is used to soften the probability distribution and facilitate more informative guidance from the teacher model. During training, several data augmentation strategies are applied, including random resized cropping, random horizontal flipping, and lighting augmentation, in order to improve model generalisation and robustness. The model with the highest validation accuracy is selected and saved as the final checkpoint for evaluation. The implementation of the proposed BiSpikeNet is carried out in Python using the PyTorch framework, and the corresponding code is made publicly available on GitHub for reproducibility.

### 4.2 BiSpikeNet classification results

To comprehensively evaluate the performance of the BiSpikeNet model in the task of identifying tea leaf pests and diseases, this study generated detailed classification reports on the test dataset. Overall, the model demonstrated stable and high classification performance across all categories. Its F1 score consistentlyremained above 0.99, exhibiting strong discriminative capability and robust generalisation ability, as shown in **Table 2**.

**Table 2:** Detection of pest and disease categories.

Tea categories	Precision	Recall	F1-score	Support
Broken tea leaves	0.9875	0.9875	0.9875	23
Tea mosquito blight	0.9874	0.9737	0.9867	119
Tea leaf brown spot disease	0.9921	0.9806	0.9851	68
Tea leaf red spot disease	0.9782	0.9655	0.9765	97
Tea leaf grey spot disease	0.9879	0.9781	0.9830	97
**Tea leaf spot disease**	**0.9992**	**0.9992**	**0.9992**	**94**
Healthy Tea	0.9936	0.9936	0.9936	112

Bold values indicate the highest model accuracy values.

Specifically, this model achieved optimal performance in the tea leaf spot disease category. Its precision, recall, and F1 score all reached 0.9992, indicating near-perfect classification results. Similarly, it demonstrated outstanding performance across categories including damaged tea leaves, tea mosquito blight, and tea grey spot disease. F1 scores nearing 0.99 reflect how the dual design—combining lightweight architecture with SNN temporal augmentation and pulse residuals—endows the model with exceptional capability in both pest and disease identification tasks.

For the majority of categories with larger sample sizes, such as damaged tea leaves, the model maintains an F1 score of 0.9875. This indicates that even under conditions of high intra-class variation, the model remains capable of effective feature learning and generalisation.

However, the model exhibits a relatively low recall rate of 0.9655 for the tea leaf red spot disease category. This phenomenon may stem from subtle feature differences between categories or a limited number of representative samples. The misclassification of some positive instances indicates that the model has not yet sufficiently learned the discriminative features for this category. Future work may consider targeted data augmentation, reweighted loss functions, or category-specific attention refinement strategies to further enhance recognition performance for this category.

Overall, the model achieved F1 scores exceeding 0.97 across all seven categories, with two categories surpassing 0.99. These results fully validate the effectiveness of structural designs including lightweight architecture, the SNNMultiShortcutBlock pulse residual block, BiSNN temporal binarisation, and attention mechanisms.

### 4.3 Comparison of BiSpikeNet with contemporary models across different datasets

To further validate the effectiveness of the BiSpikeNet model, this study conducted systematic comparative experiments on both the CIFAR-10 dataset and a seven-class tea pest and disease dataset. The experimental results are presented in **Tables 3** and **4**.

**Table 3:** Experiments of binarisation methods on the CIFAR-10 dataset.

Model	Method (Year)	Bit-Width (W/A)	Acc. (%)	FLOPS (M)	Params (K)
ResNet-20	DoReFa (2016)	1/1	79.3	40.81	272.47
	DSQ (2019)	1/1	84.1	40.81	272.55
	LNS (2020)	1/1	85.8	40.81	272.49
	IR-Net (2020) NE (2021)	1/1 1/1	86.5 85.3	40.81 40.81	273.16 272.49
	SD-BNN (2022)	1/1	86.9	40.81	272.49
	BiRealNet (2025)	1/1	87.9	40.81	272.48
	**Ours (2026)**	1/1	**93.2**	**40.81**	**272.29**

Bold values indicate the highest model accuracy values.

**Table 4:** Training results of advanced models on tea pest and disease datasets.

Model (year)	Accuracy	FLOPS (M)	Params (K)
ResNet-18 (2016)	92.34%	3634.71	11191.24
YOLO-T (2023)	97.30%	12000	6000
YOLO-TEA (2023)	82.60%	14200	795.98
TSBA-YOLO (2023)	87.83%	25000	14000
Res4NetCBAM (2023)	97.83%	5200	34100
YOLOV8-RCAA (2024)	98.14%	27.23	810.29
MobileNeXtYOLOv7 (2024)	93.50%	8297	4058.63
YOLOV8-RMDA (2024)	93.04%	7395	2656
YOLO-DBD (2024)	86.80%	9800	4100
YOLOv7-DWS (2025)	92.90%	8184	3671
LiSA-MobileNetV2 (2025)	95.68%	331.27	575.06
TLDDM (2025)	97.21%	7334	2720
BiRealNet-18 (2025)	91.02%	24.76	209.67
**Ours (2026)**	**99.92%**	**22.63**	**117.75**

Bold values indicate the highest model accuracy values.

On the CIFAR-10 dataset, ResNet-20 has been widely adopted as the standard backbone network for performance comparison among existing binary methods. Following this well-established benchmarking protocol, the competing approaches—including BiRealNet, DoReFa (Zhou et al., 2016), DSQ (Gong et al., 2019), NE (Zhang et al., 2021), LNS (Han et al., 2020), IR-Net (Qin et al., 2020), and SD-BNN (Xue et al., 2022)—are uniformly evaluated under the ResNet-20 architecture to ensure a fair and equitable comparison. These models are broadly representative in image recognition tasks and thus serve as reliable references for validating the performance of BiSpikeNet on standard visual benchmarks.

To evaluate BiSpikeNet’s applicability in specific domain tasks, this study further introduced a series of lightweight models tailored for agricultural vision tasks onto a seven-class tea pest and disease dataset. These models include TLDDM (Wang et al., 2025b), YOLOv7-DWS (Song et al., 2025), YOLO-T (Soeb et al., 2023), YOLOv8-RCAA (Wang et al., 2024b), MobileNeXt-YOLOv7 (Wang and Xu, 2025), YOLOv8-RMDA (Ye et al., 2024), Res4Net-CBAM (Bhuyan et al., 2024), YOLO-DBD (Leng et al., 2024), YOLO-Tea (Xue et al., 2023), and TSBA-YOLO (Lin et al., 2023). These models represent the evolving trends in modern CNN architecture design, YOLO frameworks, and attention mechanism strategies within agricultural contexts, serving as crucial benchmarks for evaluating BiSpikeNet’s competitiveness in tea pest and disease identification tasks.

It should be noted, however, that in the subsequent application for tea-disease classification, the same ResNet-20 depth configuration is not retained. Given the complex morphology of disease lesions, the notable scale variations, and the substantial background interference present in tea-disease images, the network depth and receptive field configurations of the backbone have been deliberately tailored to facilitate more effective extraction of discriminative, fine-grained disease phenotypic features, thereby accommodating the specific demands of the real-world agricultural scenario. In summary, the CIFAR-10 experiments are primarily oriented towards algorithmic fairness verification under standardised benchmarks, whereas the tea-disease experiments are centred on feature adaptation for a specific domain task. The discrepancy in backbone structures between the two experimental setups is therefore justified by their distinct validation objectives and the inherent differences in data characteristics.

As shown in **Table 3**, the performance comparison of different binarisation methods on the CIFAR-10 dataset demonstrates that the proposed BiSpikeNet achieves significant improvements in classification accuracy. Notably, to ensure a fair and rigorous comparison, all reported methods, including DoReFa, DSQ, NE, LNS, IR-Net, SD-BNN, BiRealNet, and the proposed approach, are implemented and evaluated under strictly identical experimental settings using the same ResNet-20 architecture with 1-bit weights and 1-bit activations. Consequently, the total number of parameters differs only marginally across methods, primarily due to the introduction of learnable scaling factors at either the layer level or the channel level, whilst the computational complexity measured in FLOPs remains essentially unchanged. Such a direct and consistent comparison isolates the influence of network depth and parameter scale, thereby ensuring that any observed improvement in accuracy can be attributed solely to algorithmic advances rather than architectural differences. Under this consistent experimental setup, BiSpikeNet achieves a test accuracy of 93.20%, outperforming other mainstream binarisation methods, including DoReFa at 79.3%, DSQ at 84.1%, NE at 85.3%, LNS at 85.8%, IR-Net at 86.5%, SD-BNN at 86.9%, and BiRealNet at 87.9%.Compared with the strongest baseline, BiRealNet, the proposed method improves accuracy by approximately 5.3 percentage points, demonstrating its effectiveness and superiority in binary neural network optimization. Although BiSpikeNet is conceptually inspired by the BiRealNet framework, the comparison in this experiment is strictly conducted within a unified ResNet-20 backbone to ensure fairness and validity.

This outcome demonstrates that BiSpikeNet not only retains the advantages of binary networks in model compression and computational cost reduction but also achieves a breakthrough in representational capacity. Its performance enhancement is primarily attributable to the introduced spike neural network binary mechanism and multi-branch lightweight spike residual design. The temporal dynamic characteristics of SNN binarisation enhance the model’s ability to characterise feature structures. The three-branch SNNMultiShortcutBlock enhances gradient flow and information exchange through multi-path feature fusion, thereby maintaining robust representational capacity even at extremely low bit widths. Compared to full-precision models requiring high-accuracy computation [e.g., ResNet-18 achieves approximately94.14% accuracy (He and Garcia, 2009)], BiSpikeNet substantially reduces memory consumption and computational energy expenditure while sacrificing only minimal accuracy (approximately 0.94%). This equilibrium demonstrates its synergistic advantage in balancing lightweight architecture with high performance.

In summary, BiSpikeNet’s robust performance on the CIFAR-10 dataset validates the effectiveness of its fusion of spiking neural networks with lightweight binarisation design. This model provides a competitive solution for high-precision visual recognition tasks on low-power devices.

According to the performance comparison results of the seven-class tea pest and disease dataset shown in **Table 4**, the BiSpikeNet model proposed in this study achieved a classification accuracy of 99.92%. This accuracy significantly outperformed all listed comparison models. Concurrently, the model requires only 22.63 MFLOPs computational effort and 117.75 K parameters. This outcome demonstrates exceptionally high lightweight and efficiency advantages.

Compared to traditional lightweight architectures, BiSpikeNet achieves significant improvements in both accuracy and efficiency. For instance, ResNet-18 boasts an accuracy rate of 92.34%, yet its parameter count reaches 11.19 million with 3.63 GFLOPs, imposing a substantial computational burden. BiRealNet- 18, another binary lightweight model, achieves 91.02% accuracy while being compressed to 209.67K parameters and 24.76 MFLOPs. This accuracy remains approximately 8 percentage points lower than BiSpikeNet.

Among the YOLO-based improved models, YOLOv8-RCAA achieved a relatively high accuracy of 98.14%, yet its parameter count of 810.29 K and FLOPs of 27.23 M remain substantially higher than BiSpikeNet. Other models such as TSBA-TOLO and YOLO-DBD either fall short of 90% accuracy or exhibit significantly elevated computational overhead. The aforementioned models struggle to strike a balance between precision and lightweight design. BiSpikeNet achieves near-perfect classification performance with minimal parameters and computational cost by integrating spike neural network binarisation with multi-branch lightweight spike residual design. This model demonstrates outstanding overall competitiveness.

In summary, BiSpikeNet not only achieves the current state-of-the-art recognition accuracy in tea leaf pest and disease classification tasks, but also strikes an exceptional balance between precision and efficiency through its highly competitive lightweight architecture. Furthermore, it converges remarkably quickly, reaching stable performance within approximately 80 iterations, as illustrated in **Figure 6**. This model is particularly well-suited for lightweight, resource-efficient systems and mobile terminals, offering an efficient and reliable solution for real-time disease identification in intelligent agriculture.

**Figure 6:**
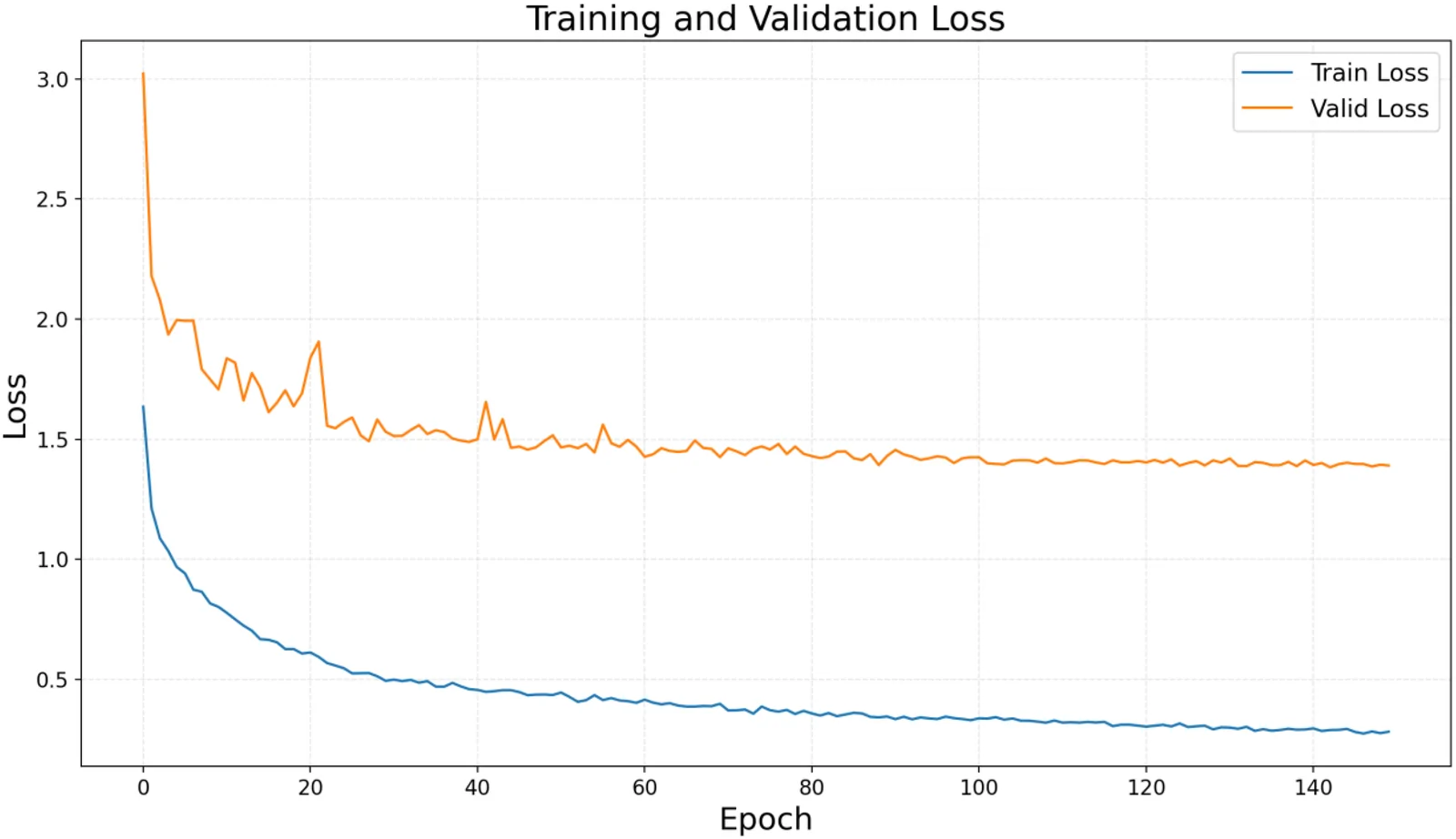
BiSpikeNet validation and loss convergence.

### 4.4 Classification accuracy and computational efficiency of ablation experiments

To systematically evaluate the independent contribution of each structural optimization scheme to the overall model performance, a series of ablation experiments were designed and executed. These experiments progressively validated the effectiveness of three design aspects: model lightweighting, the BiSNN module, and the introduction of the SNNMultiShortcutBlock pulse residual structure, as shown in **Table 5**. To ensure statistical power and reproducibility of the experimental results, each model configuration underwent a complete training and testing process under five independent random seeds. The final report presents the average classification accuracy and its standard deviation across the five repeated experiments. This experimental design effectively mitigates random fluctuations caused by parameter random initialisation, thereby more reliably revealing the true impact of each structural modification on model performance.

**Table 5:** Ablation experiments.

Model	Attention	BiSNN	MultiBlock	KD	Accuracy	Precision	Params (K)	FLOPS (M)
BiRealNet-18	×	×	×	×	91.02*±* 0.42%	90.53*±* 0.39%	209.67	24.76
LB-BiRealNet	✓	×	×	×	92.30*±* 0.40%	91.25*±* 0.38%	178. 73	23.92
BiSNNNet	✓	✓	×	×	95.82*±* 0.38%	94.23*±* 0.38%	127.83	23.06
BiMultiNet	✓	×	✓	×	95.78*±* 0.21%	94.64*±* 0.20%	148.83	23.46
BiSpikeNet	✓	✓	✓	×	99. 13*±* 0.24%	99.06*±* 0. 19%	117.75	22.63
Ours	✓	✓	✓	✓	99.92*±* 0. 15%	99.59*±* 0. 11%	117.75	22.63

Experimental results demonstrate that the lightweight architecture LB-BiRealNet achieves an average classification accuracy of 92.30 *±* 0.40%, while largely preserving the model’s representational capacity.This outcome represents a 1.28 percentage point improvement over the original BiRealNet baseline model’s 91.02%. This outcome demonstrates that rational module pruning and structural reorganisation can significantly enhance the efficiency of internal information propagation within the network. This design establishes a robust foundation for subsequent architecture enhancements.

This study employs the spiking neural network binary module BiSNN, which incorporates spiking dynamics, forming the BiSNNNet. The model achieves an average accuracy of 95.82 *±* 0.38% and a precision rate of 94.23 *±* 0.38%. The results validate The key role of BiSNNNet in enhancing feature discriminative power. While SNNs bolster temporal modelling capabilities and enrich representational space at low energy consumption, BiSNNNet occasionally exhibits abnormal accuracy loss during training.

Building upon this foundation, this study introduces a three-branch SNNMultiShortcutBlock and Adaptive-PLIF neurons into LB-BiRealNet to construct BiMultiNet. Five independent experiments demonstrate an accuracy of 95.78 ± 0.21% and a precision of 94.64 ± 0.20%, significantly surpassing LB- BiRealNet and baseline methods. This indicates that structural optimization and enhanced neural dynamics jointly improve feature representation capability, learning stability, and generalisation performance at both macro and micro levels.

To further enhance representation learning, a knowledge distillation strategy is incorporated during training, where a pretrained ResNet-18 teacher provides soft supervision through KL divergence with temperature scaling. An ablation study is conducted to evaluate its effect. The results show that BiSpikeNet achieves 99.13% accuracy without knowledge distillation, and 99.92% with knowledge distillation, indicating that knowledge distillation acts as a complementary optimization strategy that improves decision boundary refinement without changing the fundamental contribution of the proposed architecture.

Further analysis shows that BiSNNNet, which only includes the BiSNN module, and BiMultiNet, which includes the SNNMultiShortcutBlock but excludes BiSNN, exhibit complementary limitations. Specifically, BiSNNNet lacks sufficient structural feature aggregation capability, while BiMultiNet cannot fully exploit temporal spiking dynamics for discriminative representation. These observations indicate that neither component alone is sufficient to construct the complete BiSpikeNet framework.

The full BiSpikeNet integrates Attention, BiSNN, and SNNMultiShortcutBlock, achieving a final performance of 99.92% accuracy and 99.59% precision with consistent results across repeated experiments. In addition, the model maintains a low computational cost of 22.63M FLOPs and 117.75K parameters. Overall, the results demonstrate that the proposed architecture achieves a balanced improvement in representation capability and classification performance.

Comparing the training and validation loss curves of BiSpikeNet and BiRealNet, as shown in **Figure 7**, BiSpikeNet exhibits a smooth training loss curve with validation loss converging rapidly to a low level and exhibiting minimal fluctuation. In contrast, BiRealNet’s training loss persists near 3.0 for an extended period, while its validation loss declines slowly with pronounced oscillations. The results demonstrate that the spiking dynamics mechanism within the BSNN module significantly enhances the model’s convergence efficiency and training stability, exhibiting superior robustness and generalisation capabilities compared to BiMLP.

**Figure 7:**
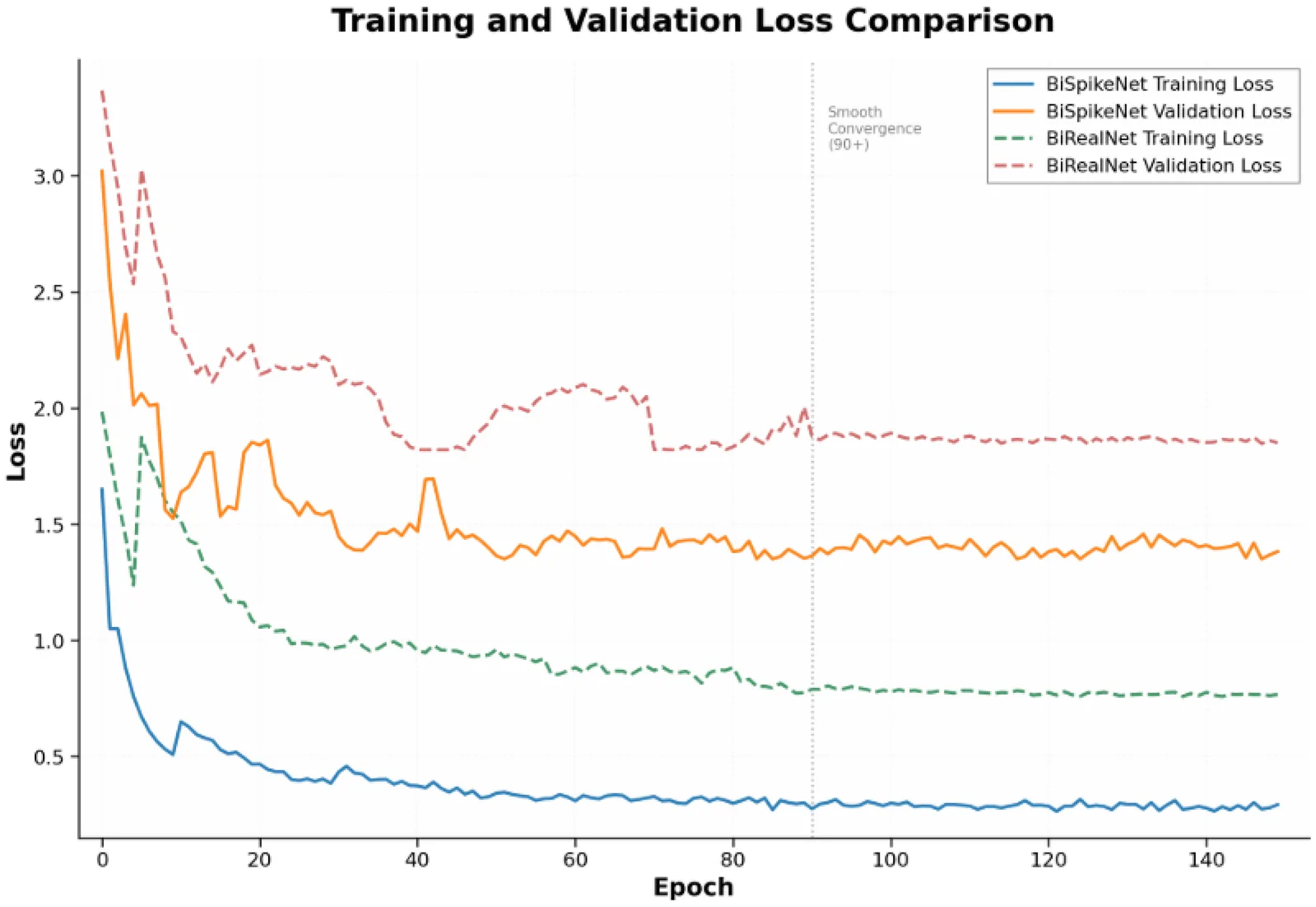
Convergence plots for BiSNN in BiSpikeNet and BiMLP in BiRealNet.

### 4.5 Visualisation analysis of pulse residual blocks following model design

To enhance the model’s interpretability and validate its effectiveness in classifying tea leaf pests and diseases, this study employed Gradient-Weighted Class Activation Map (Grad-CAM) to generate visual explanations (Fayyaz et al., 2025). Grad-CAM highlights regions within the input image that exert the greatest influence on the model’s decision by calculating the gradient of the predicted class relative to the activation of the final convolutional layer. This method generates a heatmap reflecting the model’s focus, where red indicates areas of high attention and blue denotes areas of low attention.

This study selected three representative disease categories for visualisation analysis. The chosen categories were tea mosquito blight, tea leaf grey spot disease, and damaged tea leaves. These three categories correspond to distinct classification challenges and sample distributions. **Figure 8** displays the Grad- CAM visualisation results for each category across the following models: BiRealNet, LB-BiRealNet and BiSpikeNet.

**Figure 8:**
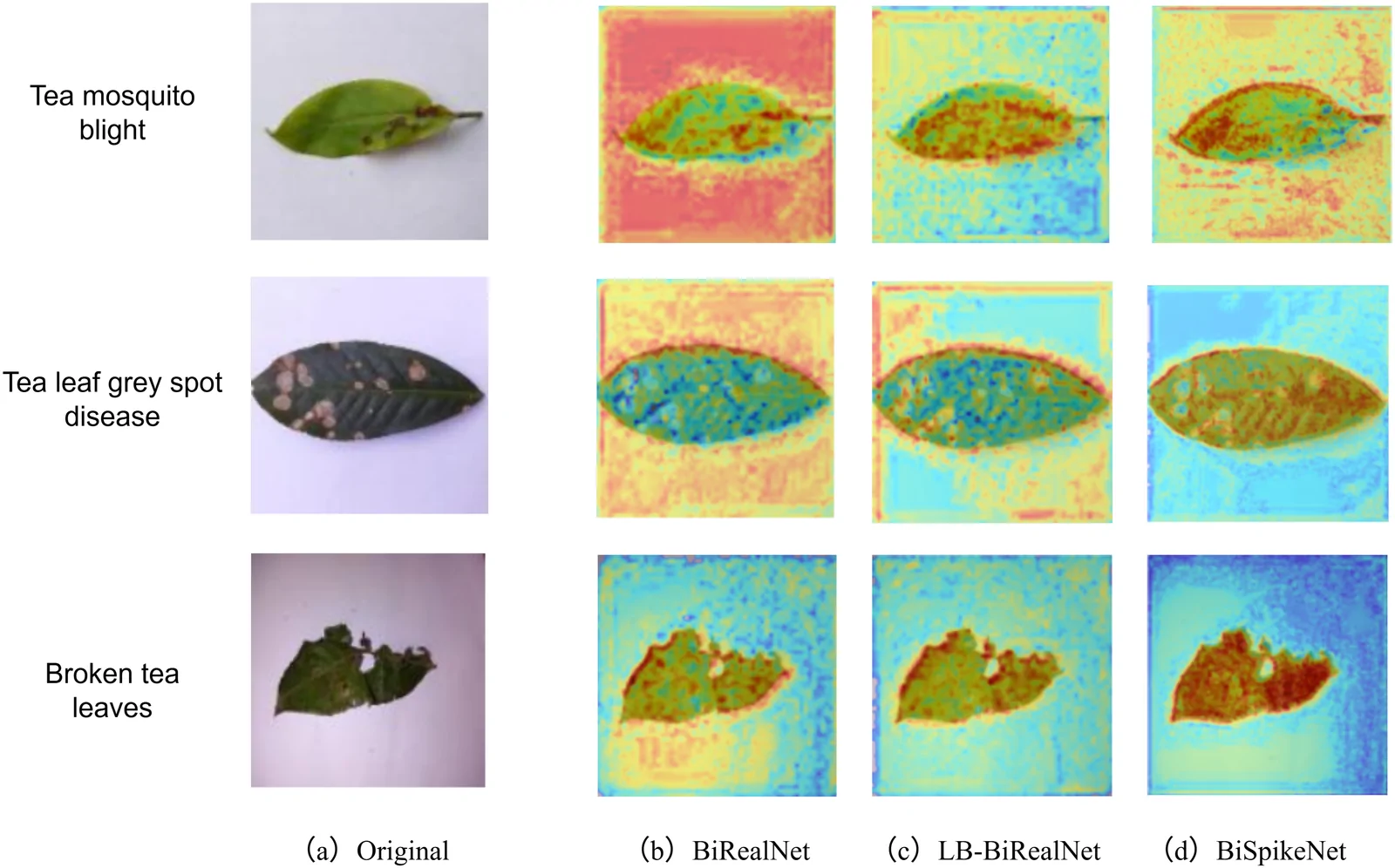
Grad-CAM visualisation of the design variant across three representative categories of tea pests and diseases. **(a)** Original. **(b)** BiRealNet. **(c)** LB-BiRealNet. **(d)** BiSpikeNet.

Analysis of the visualisation results reveals significant differences in the attention patterns of different models towards diseased areas. This variation directly reflects the effectiveness of their internal feature extraction and decision-making mechanisms. For certain disease categories, the original model exhibits a relatively dispersed attention distribution. This model fails to maintain stable focus on discriminative lesion areas. This phenomenon indicates its limited capability for feature selection within complex backgrounds. Models incorporating lightweight structures and attention mechanisms (such as LB-BiRealNet) mitigate this issue to a certain extent. This model demonstrates greater accuracy in localising lesions, exhibiting improved interpretability and classification stability.

Furthermore, upon concurrently incorporating the BiSNN module and SNNMultiShortcutBlock module into the BiSpikeNet model, the architecture demonstrated enhanced robustness and comprehensive visual comprehension capabilities. This model not only focuses on superficial structural features such as leaf margins but also integrates global contextual information to capture the overall morphological structure of tea leaves. Concurrently, BiSpikeNet exhibits high semantic awareness in identifying diseased regions. It maintains high response intensity in healthy leaf regions to construct complete object representations, whilst directing discriminative attention to diseased areas based on their pathological characteristics. As illustrated in **Figure 8**, the activation heatmaps generated by this model exhibit greater structural coherence and logical consistency in spatial distribution. These heatmaps delineate the tea leaf’s overall contour whilst precisely highlighting morphological and textural alterations in specific diseased regions.

This phenomenon demonstrates that BiSpikeNet’s binary mechanism for spiking neural networks, coupled with its multi-path lightweight spiking residual architecture, enables parallel processing of visual cues at different levels. Shallow paths preserve details and local features, while deep paths integrate semantic and global context. This design achieves acute recognition of local lesions while maintaining holistic structural perception. This “whole-part” collaborative attention mechanism not only enhances the model’s adaptability to complex agricultural scenarios but also provides more reliable classification decisions grounded in human visual cognition. This outcome signifies substantial progress in synergistically optimising visual interpretability and classification performance.

In summary, the model incorporating gradient retention mechanisms and attention structures not only demonstrates improved classification performance but also significantly enhances the model’s ability to focus on semantically relevant regions within images. This design provides more intuitive visual evidence for model decision-making.

## 5 Conclusion

This paper proposes a novel binary spike neural network architecture termed BiSpikeNet. The core innovation of this model lies in the introduction and implementation of a “Diagnostic” design methodology, wherein the visualisation and quantitative analysis of internal feature maps serve as the fundamental basis for guiding its overall architectural design. Through systematic diagnostics of intermediate feature maps’ semantic distribution, response intensity, and information flow pathways, we precisely pinpointed critical bottlenecks in capturing temporal patterns and spatial details. This led to the original design of novel binary spiking information processing units and a multi-temporal short-circuit connection structure. The former directly stems from analyses of feature response sparsity and event-driven efficiency, while the latter constitutes a targeted solution to optimise deep feature flow propagation pathways. Concurrently, the model incorporates a dynamic feature calibration mechanism, wherein its weight parameters are likewise learned from spatial attention heatmaps of feature maps. This closed-loop paradigm of “observation- diagnosis-design” ensures that every structural innovation directly addresses specific challenges in feature representation. Consequently, it achieves profound synergy between the biomimetic dynamics of spiking neural networks, the ultimate efficiency of binarisation, and the visual demands of the task. Ultimately, this approach constructs a unified architecture that simultaneously delivers high accuracy, low computation waste, efficient resource usage, and robust temporal modelling capabilities.

Experimental evaluations thoroughly validate the efficacy of the original BiSpikeNet architecture. On specialised tea leaf image test datasets encompassing diverse complex scenarios, the model achieves state-of-the-art average detection accuracy, significantly outperforming multiple mainstream baseline models. In terms of efficiency, BiSpikeNet demonstrates notable advantages in key metrics such as parameter count and inference latency, successfully achieving the design objective of “high accuracy with low computational overhead”. Systematic comparative analysis further indicates that our proposed pulse- residual collaborative mechanism can more precisely capture the microscopic morphological variations and spatio-temporal evolution characteristics of tea leaf lesions. Consequently, it learns more discriminative feature representations under stringent lightweight constraints. Although the current work validates the effectiveness of BiSpikeNet through comprehensive software simulations, deployment on edge-computing and neuromorphic hardware platforms remains ongoing work. Future studies will focus on real-device implementation and optimization to further verify the practical advantages of the proposed architecture in terms of latency, power consumption, and deployment efficiency.

Despite the positive progress achieved by BiSpikeNet, future work will be pursued in the following directions to address current limitations and practical application requirements:

Firstly, enhance the model’s adaptability in extreme and unstructured environments. To address complex scenarios involving variable lighting, diverse crop varieties, and varying planting densities, future research will focus on open-world adaptive perception technologies. This includes developing agricultural scenario simulation engines to generate diverse synthetic data. Designing test-time adaptation mechanisms will enable models to rapidly adjust using online imagery. Explore domain-invariant feature learning methods to enhance intrinsic robustness.

Secondly, establish a large-scale, fine-grained, open dataset on tea plant growth and health. To overcome data limitations, we shall promote the collaborative development of a community-driven, standardised benchmark dataset. This will encompass a broader range of varieties, more detailed annotations of disease progression, and richer environmental context, thereby providing a foundation for model refinement and sector advancement.

Thirdly, explore further model compression, hardware deployment, and resource-efficient optimization to support extremely lightweight inference. Although the proposed BiSpikeNet has demonstrated promising performance in software-based simulations, practical deployment on edge-computing and neuromorphic hardware remains an important next step. The current study primarily focuses on the theoretical design and algorithmic validation of the proposed architecture. Future research will investigate the deployment of BiSpikeNet on resource-constrained edge platforms and neuromorphic processors to evaluate real-world inference latency, power efficiency, and hardware compatibility. In addition, hierarchical knowledge distillation, mixed-precision computation, and dynamic sparsification strategies will be explored to further improve computational economy and energy efficiency. Such efforts will help bridge the gap between simulation-based evaluation and practical intelligent agricultural applications.

Furthermore, we shall explore intelligent agricultural systems featuring closed-loop multimodal perception and decision-making. Future endeavours will focus on deeply integrating BiSpikeNet’s temporal visual capabilities with multispectral/hyperspectral imaging, near-surface remote sensing, meteorological and soil data, and agronomic knowledge graphs. This will establish a closed-loop system encompassing “perception-diagnosis-decision-control”, enabling a leap from pest and disease identification to trend forecasting and precision regulation. Such advancements will serve precision agriculture and sustainable development.

## Data Availability

The datasets presented in this study can be found in online repositories. The names of the repository/repositories and accession number(s) can be found in the article/supplementary material.

## References

[B1] Bhuyan P. Singh P. K. Das S. K. (2024). Res4net-cbam: a deep cnn with convolution block attention module for tea leaf disease diagnosis. Multimedia Tools Appl. 83, 48925–48947. doi: 10.1007/s11042-023-17472-630311153

[B2] Bulat A. Martinez B. Tzimiropoulos G. (2021). “ High-capacity expert binary networks”, in: 9th International Conference on Learning Representations (ICLR), (Virtual Event: OpenReview.net), ArXiv:2010.03558.

[B3] Bunkhumpornpat C. Boonchieng E. Chouvatut V. Lipsky D. (2024). Flex-smote: synthetic over-sampling technique that flexibly adjusts to different minority class distributions. Patterns 5, 101073. doi: 10.1016/j.patter.2024.101073 39568474 PMC11573909

[B4] Chen Z. M. Luo Z. X. (2025). Management of insect pests on tea plantations: safety, sustainability, and efficiency. Annu. Rev. Entomol. 70, 359–377. doi: 10.1146/annurev-ento-013024-014757 39383439

[B5] Duhan S. Gulia P. Gill N. S. Shukla P. K. Khan S. B. Almusharraf A. . (2024). Investigating attention mechanisms for plant disease identification in challenging environments. Heliyon 10, e29802. doi: 10.1016/j.heliyon.2024.e29802 38707335 PMC11066637

[B6] Fayyaz A. M. Abdulkadir S. J. Talpur N. Al-Selwi S. M. Hassan S. U. Sumiea E. H. (2025). Grad-cam (gradient-weighted class activation mapping): a systematic literature review. Comput. Biol. Med. 198, 111200. doi: 10.1016/j.compbiomed.2025.111200 41108904

[B7] Gong R. Liu X. Jiang S. Li T. Hu P. Lin J. . (2019). “ Differentiable soft quantization: bridging full-precision and low-bit neural networks”, in: Proceedings of the IEEE/CVF International Conference on Computer Vision (ICCV), (Seoul: IEEE), 4852–4861.

[B8] Guo Y. Peng W. Chen Y. Zhang L. Liu X. Huang X. . (2023). Joint a-snn: joint training of artificial and spiking neural networks via self-distillation and weight factorization. Pattern Recognit. 142, 109639. doi: 10.1016/j.patcog.2023.10963942574925

[B9] Han K. Wang Y. Xu Y. Xu C. Wu E. Xu C. (2020). “ Training binary neural networks through learning with noisy supervision Pmlr”, in: International Conference on Machine Learning (ICML), (Virtual: PMLR), 4017–4026.

[B10] He H. Garcia E. A. (2009). Learning from imbalanced data. IEEE Trans. Knowl. Data Eng. 21, 1263–1284. doi: 10.1109/TKDE.2008.23925079929

[B11] He K. Zhang X. Ren S. Sun J. (2016). “ Deep residual learning for image recognition”, in: Proceedings of the IEEE Conference on Computer Vision and Pattern Recognition (CVPR), (Las Vegas, NV: IEEE), 770–778.

[B12] He Y. Zhang L. Wu W. Zhou H. (2024). Binarizing by classification: is soft function really necessary? IEEE Trans. Circuits Syst. Video Technol. 34, 973–982. doi: 10.1109/TCSVT.2023.328857225079929

[B13] Le V. S. Herrmann L. Brau L. Lesueur D. (2023). Sustainable green tea production through agroecological management and land conversion practices for restoring soil health, crop productivity and economic efficiency: evidence from northern Vietnam. Soil Use Manage. 39, 1185–1204. doi: 10.1111/sum.1288540046247

[B14] Leng X. Chen J. Huang J. Zhang L. Li Z. (2024). An improved yolov8-based method for real-time detection of harmful tea leaves in complex backgrounds. Phyton-International J. Exp. Bot. 93, 2963–2981. doi: 10.32604/phyton.2024.057166

[B15] Li X. Sun W. Ji Y. Huang W. (2026). A plot-to-track association framework based on graph representation learning for compact hfswr. IEEE Trans. Aerosp. Electron. Syst. 62, 12742–12760. doi: 10.1109/taes.2026.370338525079929

[B16] Li Y. Zhang H. Zhao L. Wang J. (2024). Tea leaf disease recognition based on convolutional neural network and transfer learning. Comput. Electron. Agric. 218, 108620. doi: 10.1016/j.compag.2024.10862042574925

[B17] Lin J. Bai D. Xu R. Lin H. (2023). Tsba-yolo: an improved tea diseases detection model based on attention mechanisms and feature fusion. Forests 14, 619. doi: 10.3390/f1403061930654563

[B18] Lin C. Hu X. Zhan Y. Hao X. (2024). Mobilenetv2 with spatial attention module for traffic congestion recognition in surveillance images. Expert Syst. Appl. 255, 124701. doi: 10.1016/j.eswa.2024.12470142574925

[B19] Nardini F. M. Rulli C. Trani S. Venturini R. (2025). Neural network compression using binarization and few full-precision weights. Inf. Sci. 716, 122251. doi: 10.1016/j.ins.2025.12225142574925

[B20] Pruteanu M. A. Ungureanu N. Vladut¸ V. Matache M.-G. Nit¸u M. (2023). Contributions to the optimization of the medicinal plant sorting process into size classes. Agriculture 13, 645. doi: 10.3390/agriculture1303064530654563

[B21] Qin H. Gong R. Liu X. Shen M. Wei Z. Yu F. . (2020). “ Forward and backward information retention for accurate binary neural networks”, in: Proceedings of the IEEE/CVF Conference on Computer Vision and Pattern Recognition (CVPR), (Seattle, WA: IEEE), 13999–14008.

[B22] Rodrguez-Lira D. C. Crdova-Esparza D. M. lvarez Alvarado J. M. Terven J. Romero-Gonzlez J. A. Rodrguez-Resndiz J. (2024). Trends in machine and deep learning techniques for plant disease identification: a systematic review. Agriculture-Basel 14, 2188. doi: 10.3390/agriculture1412218830654563

[B23] Rodriguez-Sanchez J. Martins Da Silva R. Chu Y. Rodriguez L. Zhang J. Johnsen K. . (2025). Aerial imagery and segment anything model for architectural trait phenotyping to support genetic analysis in peanut breeding. Plant Phenomics 7, 100126. doi: 10.1016/j.plaphe.2025.100126 42040996 PMC13109298

[B24] Roy C. Naskar S. Ghosh S. Rahaman P. Mahanta S. Sarkar N. . (2024). Sucking pest management in tea (camellia sinensis (l.) kuntze) cultivation: integrating conventional methods with bio-control strategies. Crop Prot. 183, 106759. doi: 10.1016/j.cropro.2024.10675942574925

[B25] Sajitha P. Andrushia A. D. Anand N. Lubloy E. (2026). Channel-attentive yolov5 and capsule auto-encoder for pomegranate disease detection. Egypt. Inf. J. 33, 100877. doi: 10.1016/j.eij.2025.10087742574925

[B26] Shahadat N. Maida A. S. (2025). Analyzing parameter-efficient convolutional neural network architectures for visual classification. Sensors 25, 7663. doi: 10.3390/s25247663 41471657 PMC12736689

[B27] Shen X. Sun R. Sui G. Xu R. Zhou X. Yan D. . (2026). Downscaling prediction of urban wind environment based on pbms and hybrid mlp-cnn model. Urban Clim. 65, 102747. doi: 10.1016/j.uclim.2025.10274742574925

[B28] Shrisha H.-S. Anupama V. (2025). Multinet: a lightweight deep learning group of models for fruit maturity detection. Measurement: Digitalization 4, 100012. doi: 10.1016/j.meadig.2025.10001242574925

[B29] Soeb M. J. A. Jubayer M. F. Tarin T. A. Al Mamun M. R. Ruhad F. M. Parven A. . (2023). Tea leaf disease detection and identification based on yolov7 (yolo-t). Sci. Rep. 13, 6078. doi: 10.1038/s41598-023-33270-4 37055480 PMC10102080

[B30] Song J. Zhang Y. Lin S. Han H. Yu X. (2025). Tlddm: an enhanced tea leaf pest and disease detection model based on yolov8. Agronomy-Basel 15, 727. doi: 10.3390/agronomy1503072730654563

[B31] Wang S. Dong S. Chen S. Liu M. (2025a). An efficient scale-aware model based on the improved rt-detr for pomegranate growth stage detection. Neurocomputing 647, 130462. doi: 10.1016/j.neucom.2025.13046242574925

[B32] Wang J. Li M. Han C. Guo X. (2024b). Yolov8-rcaa: a lightweight and high-performance network for tea leaf disease detection. Agriculture-Basel 14, 1240. doi: 10.3390/agriculture1408124030654563

[B33] Wang H. Sun S. Ren P. (2023). Meta underwater camera: a smart protocol for underwater image enhancement. ISPRS J. Photogramm. Remote Sens. 195, 462–481. doi: 10.1016/j.isprsjprs.2022.12.00742574925

[B34] Wang X. Wu Z. Xiao G. Han C. Fang C. (2025b). Yolov7-dws: tea bud recognition and detection network in multi-density environment via improved yolov7. Front. Plant Sci. 15, 1503033. doi: 10.3389/fpls.2024.1503033 39840356 PMC11747160

[B35] Wang J. Xu K. (2025). An accelerating method of yolov7 based on lightweight network architecture. Appl. Sci.-Basel 15, 2528. doi: 10.3390/app1505252830654563

[B36] Wang Y. Yang X. Li Z. Liu H. (2025c). Tea disease identification based on deep learning and improved attention mechanism. Comput. Electron. Agric. 229, 109915. doi: 10.1016/j.compag.2025.10991542574925

[B37] Wang H. Zhang W. Ren P. (2024a). Self-organized underwater image enhancement. ISPRS J. Photogramm. Remote Sens. 215, 1–14. doi: 10.1016/j.isprsjprs.2024.06.01942574925

[B38] Wang Y. Zhang T. Wu X. Li Q. Li Y. Wang Q. (2025d). Tpdnet: triple phenotype deepen networks for monocular 3d object detection of melons and fruits in fields. Plant Phenomics 7, 100048. doi: 10.1016/j.plaphe.2025.100048 41415180 PMC12709957

[B39] Wang H. Zhang W. Xu Y. Li H. Ren P. (2026). Watercyclediffusion: visual–textual fusion empowered underwater image enhancement. Inf. Fusion 127, 103693. doi: 10.1016/j.inffus.2025.10369342574925

[B40] Wang Y. F. Zhang H. Y. Zeng X. L. Wang B. W. Li W. Ding W. R. (2025e). Binary lightweight neural networks for arbitrary scale super-resolution of remote sensing images. IEEE Trans. Geosci. Remote Sens. 63, 5609716. doi: 10.1109/TGRS.2025.352969625079929

[B41] Xue P. Lu Y. Chang J. Wei X. Wei Z. (2022). Self-distribution binary neural networks. Appl. Intell. 52 (12), 13870–13882. doi: 10.1007/s10489-022-03348-z30311153

[B42] Xue Z. Xu R. Bai D. Lin H. (2023). Yolo-tea: a tea disease detection model improved by yolov5. Forests 14, 415. doi: 10.3390/f1402041530654563

[B43] Ye R. Shao G. He Y. Gao Q. Li T. (2024). Yolov8-rmda: lightweight yolov8 network for early detection of small target diseases in tea. Sensors 24, 2896. doi: 10.3390/s24092896 38733002 PMC11086262

[B44] Zhang W. Chen Y. Wang Q. Q. Chen J. (2025b). Researches on the tender leaf identification and mechanically perceptible plucking finger for high-quality green tea. J. Sci. Food Agric. 105, 2169–2178. doi: 10.1002/jsfa.13987 39472285

[B45] Zhang S. Ge F. Ding R. Liu H. Zhou X. (2021). “ Learning to binarize convolutional neural networks with adaptive neural encoder”, in: 2021 International Joint Conference on Neural Networks (IJCNN) (Shenzhen: IEEE), 1–8.

[B46] Zhang M. Y. Liu C. F. Li Z. H. Yin B. Q. (2025a). From convolutional networks to vision transformers: evolution of deep learning in agricultural pest and disease identification. Agronomy-Basel 15, 1079. doi: 10.3390/agronomy1505107930654563

[B47] Zhang Z. Lu Y. Zhao Y. Pan Q. Jin K. Xu G. . (2023). Ts-yolo: an all-day and lightweight tea canopy shoots detection model. Agronomy 13, 1411. doi: 10.3390/agronomy1305141130654563

[B48] Zhao Q. H. Wang F. W. Wang W. M. Zhang T. X. Wu H. D. Ning W. J. (2025). Research on intrusion detection model based on improved mlp algorithm. Sci. Rep. 15, 5159. doi: 10.1038/s41598-025-89798-0 39939428 PMC11821824

[B49] Zhong M. Wei L. Mo H. (2025). Research on lightweight detection of cotton leaf diseases based on self-supervised contrastive representation learning. J. Integr. Agric. doi: 10.1016/j.jia.2025.12.041

[B50] Zhou S. Wu Y. Ni Z. Zhou X. Wen H. Zou Y. (2016). Dorefa-net: Training low bitwidth convolutional neural networks with low bitwidth gradients. CoRR, abs/1606.06160. doi: 10.48550/arXiv.1606.06160

